# Macrophage lineage cells-derived migrasomes activate complement-dependent blood-brain barrier damage in cerebral amyloid angiopathy mouse model

**DOI:** 10.1038/s41467-023-39693-x

**Published:** 2023-07-04

**Authors:** Mengyan Hu, Tiemei Li, Xiaomeng Ma, Sanxin Liu, Chunyi Li, Zhenchao Huang, Yinyao Lin, Ruizhen Wu, Shisi Wang, Danli Lu, Tingting Lu, Xuejiao Men, Shishi Shen, Huipeng Huang, Yuxin Liu, Kangyu Song, Banghao Jian, Yuxuan Jiang, Wei Qiu, Quentin Liu, Zhengqi Lu, Wei Cai

**Affiliations:** 1 Department of Neurology, Mental and Neurological Disease Research Center, The Third Affiliated Hospital of Sun Yat-sen University, Guangzhou, 510630 China; 2 Guangdong Provincial Key Laboratory of Brain Function and Disease, Guangzhou, 510630 China; 3 Center of Clinical Immunology, Mental and Neurological Disease Research Center, The Third Affiliated Hospital of Sun Yat-sen University, Guangzhou, 510630 China; 4 Department of Neurosurgery, The Third Affiliated Hospital of Sun Yat-Sen University, Guangzhou, 510630 China; 5 Sun Yat-sen University Cancer Center, State Key Laboratory of Oncology in South China, Guangzhou, 510060 China

**Keywords:** Neurovascular disorders, Monocytes and macrophages, Complement cascade

## Abstract

Accumulation of amyloid beta protein (Aβ) in brain vessels damages blood brain barrier (BBB) integrity in cerebral amyloid angiopathy (CAA). Macrophage lineage cells scavenge Aβ and produce disease-modifying mediators. Herein, we report that Aβ40-induced macrophage-derived migrasomes are sticky to blood vessels in skin biopsy samples from CAA patients and brain tissue from CAA mouse models (Tg-SwDI/B and 5xFAD mice). We show that CD5L is packed in migrasomes and docked to blood vessels, and that enrichment of CD5L impairs the resistance to complement activation. Increased migrasome-producing capacity of macrophages and membrane attack complex (MAC) in blood are associated with disease severity in both patients and Tg-SwDI/B mice. Of note, complement inhibitory treatment protects against migrasomes-mediated blood-brain barrier injury in Tg-SwDI/B mice. We thus propose that macrophage-derived migrasomes and the consequent complement activation are potential biomarkers and therapeutic targets in CAA.

## Abstract (web summary)

Migrasomes are recently discovered extracellular vesicles that are produced during cellular migration. Here, the authors show that macrophage-derived migrasomes are implicated in the progression of cerebral amyloid angiopathy (CAA) through increased complement signaling using skin biopsies from CAA patients and CAA mouse models.

## Introduction

Cerebral amyloid angiopathy (CAA) is the leading cause of cognitive impairment and intracerebral heamerrhage (ICH) in the elderly^1^. As one of the most prevalent cerebral small vessel diseases (CSVD), sporadic CAA is found to be present in >50% of individuals over the age of 80 years^1^. In spite of the high incidence and detrimental prognosis, our understanding of CAA pathophysiology is still limited, resulting in the lack of effective treatment.

Deposition of fibrillar amyloid beta protein (Aβ) within small vessels and capillaries, especially the beta amyloid peptide 1-40 (Aβ40), is the signature characteristic of CAA^2–5^. The phagocytic behavior of macrophage lineage cells which include yolk sac-derived microglia, brain-resident perivascular macrophages (PVM), and circulating monocyte (Ly6C^hi^)-derived infiltrated macrophages, is critical for Aβ clearance^6–8^. Impaired macrophage scavenging activities has been proved to exacerbate CAA outcomes^9^. On the other hand, macrophage lineage cells that are overwhelmed with phagocytic cargo are found to obtain tissue destructing property^8^. The pathological impacts of Aβ-loaded macrophages in CAA are to be investigated.

Migrasomes are recently discovered extracellular vesicles that are produced during cellular migration. They contain uncertain amount of small vesicles and are linked to the originating cells through retraction fibers^10^. As continuous migrating cells, immune cells are found to be the major migrasome producers in vivo^11^. It is reported that neutrophils dispose damaged mitochondria through releasing migrasomes to maintain cell survival^11^. Although migrasome generation by macrophages has been recorded^12^, the physiological and pathological roles of macrophage-derived migrasomes remain to be elusive.

In the present study, we report that macrophage-derived migrasomes are implicated in CAA progression. Using skin samples from CAA patients and brain tissue from CAA murine models, we demonstrate that migrasomes derived from Aβ-stimulated macrophages attach to endothelial cells. Complement activation associated molecules including CD5 antigen like (CD5L) are packed into migrasomes and are thus docked on the blood vessel wall. Enrichment of CD5L in blood vessels results in severe complement dependent cytotoxicity (CDC) and subsequent blood-brain barrier (BBB) injury in Tg-SwDI/B mice. Complement inhibitory treatment protects against BBB injury in Tg-SwDI/B mice.

## Results

### Migrasomes derived from macrophages are implicated in CAA progression

**Fig. 1:**
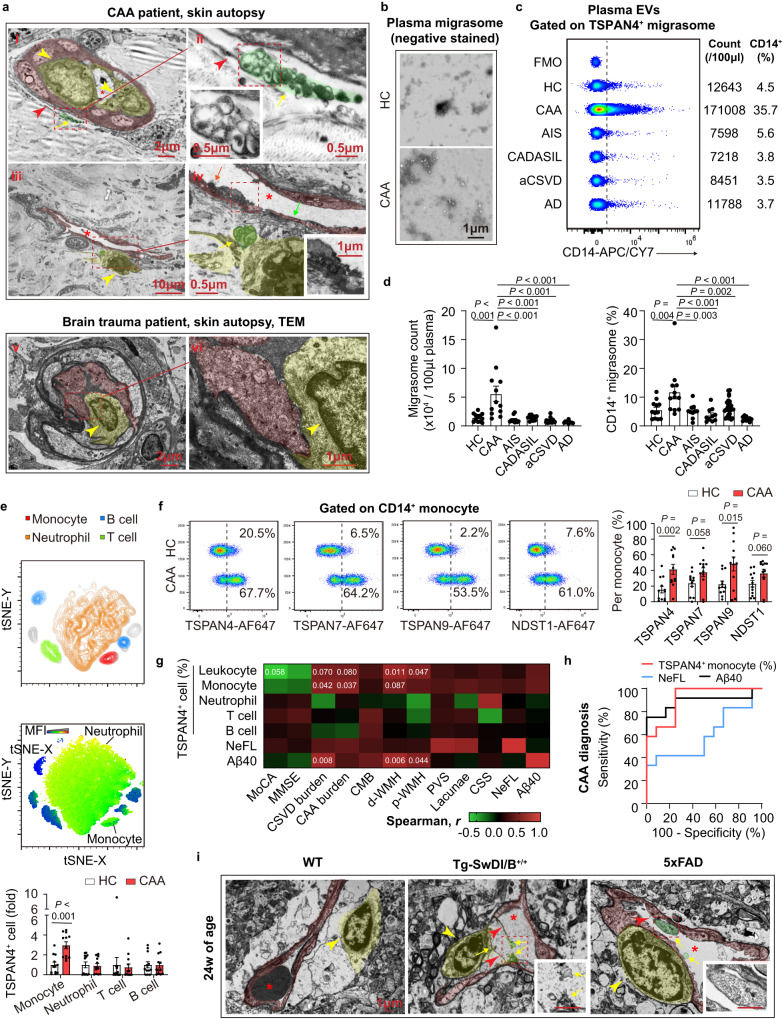
Macrophage derived migrasomes are detected in CAA patients and animal models. **a** Skin autopsy samples of a CAA patient and a brain trauma patient were subjected to TEM. Red stars emphasize blood vessel lumen. Data are representative of 3 biologically independent experiments. **b** Representative TEM images of plasma migrasomes after negative staining. Data are representative of 3 biologically independent experiments. **c**, **d** Quantity of the percentage of CD14^+^ migrasomes (per 100 μl plasma) were recorded. Plasma samples of 12 CAA patients, 10 AIS patients, 10 CADASIL patients, 28 aCSVD patients, 10 AD patients and 12 healthy controls were analyzed. Data are presented as mean values ± SEM with the indicated significance (by one-way ANOVA followed by Tukey’s post-test). **e** Upper: Representative tSNE plot of the cellular components in circulating leukocytes. Middle: tSNE analysis of TSPAN4 expression among leukocytes. Fluorescence of CD66b, CD14, CD3 and CD19 was included in the tSNE analysis. Lower: Comparison of TSPAN4 expression among leukocytes. Data are presented as mean values ± SEM with the indicated significance (by two-tailed Student’s *t* test). *N* = 12 in CAA group. *N* = 12 in HC group. **f** Migrasome markers expression in monocytes (CD14^+^). *N* = 12 in CAA group. *N* = 12 in HC group. Data are presented as mean values ± SEM with the indicated significance (by two-tailed Student’s *t* test). **g** Association of CAA biomarker candidates and CAA indications was evaluated with two-tailed Spearman correlation analysis without adjustment. The values represent *P* values, and the colors represent the values of *r*. *N* = 12 in CAA group. *N* = 12 in HC group. **h** Graph depicts the results of the ROC analysis for CAA diagnostic efficacy of monocyte TSPAN4 expression, plasma Aβ40 level and plasma NeFL concentration. TRUE = CAA, FALSE = HC. *N* = 12 in CAA group. *N* = 12 in HC group. **i** Representative images of TEM with brain tissue of CAA and wild type (WT) mice. Red stars emphasize blood vessel lumen. Data are representative of 3 biologically independent experiments. Scale bar = 1 μm. Source data are provided as a Source Data file.

To examine the pathological alterations of blood vessel in CAA patients, skin autopsy samples were analyzed with transmission electron microscopy (TEM) for the reason that brain biopsy is not included in the routine diagnostic procedures. Unexpectedly, migrasomes was observed to deposit around skin blood vessels (Fig. 1a, emphasized with yellow arrow, green tint). The migrasomes were large oval vesicles with diameter of 0.5-3.0μm and contained unequal amount of small vesicles, which coincided with the reported morphology^10^. Notably, the migrasomes seemed to be derived from macrophages that accumulated in blood vessels (Fig. 1a, ii and iv, yellow arrow head, yellow tint). In patient with brain trauma, migrasome was not evident in skin blood vessel (Fig. 1a, v and vi). Blood vessel wall of CAA patients was attached with numerous small vesicles, which were probably released from migrasomes. Endothelial cells bounded by the small vesicles displayed rough and obscure structure (Fig. 1a, iv, orange arrow, red tint) while those without small vesicles attachment showed intact appearance (Fig. 1a, iv, green arrow). In CAA patients, amyloidosis of blood vessels was evident in both the central nervous system as well as the periphery, indicating that the pathological alteration in brain blood vessels could be revealed by the manifestation of peripheral blood vessels. Therefore, we inferred that macrophage-derived migrasomes contributed to brain blood vascular disruption in CAA.

For further observation, extracellular vesicles in plasma of CAA patients and healthy donors (Healthy control, HC; age- and sex-matched) were collected and subjected to TEM analysis after negative staining. Migrasomes were observed in plasma of both HC and CAA patients but the abundance evidently increased in CAA plasma (Fig. 1b). With flow cytometric analysis, we recorded that CAA patients had up-regulated plasma migrasome counts (Fig. 1c, d, gating strategy is displayed in Fig. S1a). In accordance, peripheral leukocytes of CAA patients showed elevated expression of migrasome markers (Fig. S1b, c), which revealed active migrasome productivity. Interestingly, migrasome quantity maintained stable in patients with Alzheimer’s disease (AD) in absence of neuroimaging evidence for CAA and several other cerebral vascular disorders including acute ischemic stroke (AIS), cerebral autosomal dominant arteriopathy with subcortical infarcts and leukoencephalopathy (CADASIL) and atherosclerotic CSVD (aCSVD) (Fig. 1c, d). To be noticed, the percentage of CD14^+^ migrasomes was specifically up-regulated in CAA plasma but not in other recruited patients (Fig. 1d). Besides, in situ immunostaining revealed the attachment of migrasomes (TSPAN4^+^) produced by macrophages (CD68^+^) to blood vessels (CD31^+^) (Fig. S1d).

With flow cytometric analysis, we found that monocytes of CAA patients displayed increased expression of TSPAN4, TSPAN7, TSPAN9 and NDST1 compared with those of HC (Fig. 1e, f). In comparison, expression of migrasome markers in neutrophils, T cells and B cells was comparable between CAA patients and HC (Figs. 1e and S1e). We thus inferred that it was macrophage lineage cells that contributed to the excessive migrasome production in CAA. We next evaluated the clinical implications of macrophage-derived migrasomes. With Spearman correlation analysis, we found that TSPAN4 expression of peripheral blood monocytes was associated with decreased cognitive functions (Figs. 1g and S1f, g) and detrimental magnetic resonance imaging (MRI) indications of CAA patients (Fig. 1g). In contrast, TSPAN4 level in other immune cells displayed weak association with CAA outcomes (Fig. 1g). In receiver operating characteristic (ROC) analysis to discriminate CAA patients (TRUE) from HCs (FALSE), the parameter of TSPAN4^+^ monocyte percentage displayed considerable predictive efficiency with area under curve (AUC) of 0.9097 ± 0.0590 (*P* = 0.0007), which was higher than that of plasma NeFL (neurofilament, light polypeptide, a marker of neuroaxonal degeneration) (AUC = 0.5972 ± 0.1210, *P* = 0.4189) and Aβ40 concentration (AUC = 0.8889 ± 0.07778, *P* = 0.0012) (Fig. 1h). In CAA murine models of Tg-SwDI/B and 5xFAD mice (24w of age), deposition of macrophage-derived migrasomes was evident in brain blood vessels (Figs. 1i and S2a). Typical migrasome structures (yellow arrow) which were linked to retraction fibers (red arrow head) were revealed by TEM (Fig. 1i). Active production of TSPAN4^+^ migrasomes in CAA brains by Iba1^+^ microglia/macrophages and the attachment of migrasomes to brain blood vessels (CD31^+^) were confirmed with immunostaining (Fig. S2a). Of note, Spearman correlation analysis revealed that TSPAN4 expression in Iba1^+^ cells in mice coronal brain sections were positively correlated with that in circulating F4/80^+^ cells (Fig. S2b, c). However, elevated plasma NeFL of Tg-SwDI/B mice was associated with the increment of TSPAN4^+^F4/80^+^ cells in blood rather than TSPAN4^+^Iba1^+^ cells in brain (Fig. S2d). Our data suggest that migrasomes derived from macrophage lineage cells are implicated in CAA progression.

### Amyloid protein beta 1-40 (Aβ40) is a fundamental inducer of macrophage-derived migrasomes

**Fig. 2:**
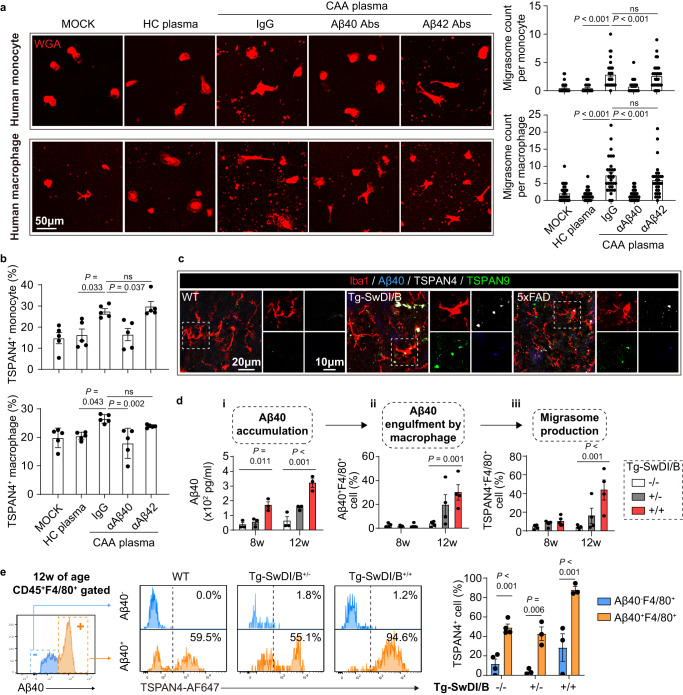
Aβ40 phagocytosis elicits migrasome production in macrophages. **a**, **b** Human monocytes were isolated from healthy donors. Human macrophages were induced with monocytes with human serum and MCSF. CAA plasma with or without Aβ40/Aβ42 removal or HC plasma was treated to monocytes/macrophages with a concentration of 10% in culture system for 6 h. **a** Immunolabeling of WGA to display migrasome production. Migrasomes of 30 cells in each group were subjected to the analysis. Data are presented as mean values ± SEM with the indicated significance (by one-way ANOVA followed by Tukey’s post-test). **b** Expression of TSPAN4 in monocytes/macrophages after plasma treatment as assessed with flow cytometry. Samples from 5 individuals in each group were subjected to the analysis. Data are presented as mean values ± SEM with the indicated significance (by one-way ANOVA followed by Tukey’s post-test). **c** Coronal brain sections from Tg-SwDI/B^+/+^ and 5xFAD mice or age- and sex-matched WT mice (24w of age) were collected and subjected to immunostaining of Iba1, Aβ40 and TSPAN4 and TSPAN9. Brain slices from 4 independent mice were subjected to the experiments. Representative images were displayed. **d**, **e** Tg-SwDI/B^+/+^, Tg-SwDI/B^+/-^ and WT mice were sacrificed at indicated age. (**d-i**) Plasma Aβ40 concentration was assessed with ELISA. **d-ii, iii** Leukocytes of mice were isolated and subjected to flow cytometric analysis of Aβ40 engulfment (CD45^+^F4/80^+^Aβ40^+^) **d-ii** and TSPAN4 expression (CD45^+^F4/80^+^TSPAN4^+^) in macrophages (**d-iii**). *N* = 3 in Tg-SwDI/B^+/+^ group, *N* = 3 in Tg-SwDI/B^+/-^ group, *N* = 4 in WT group. Data are presented as mean values ± SEM with the indicated significance (compared with WT by one tail one-way ANOVA followed by Tukey’s post-test). **e** Comparison of TSPAN4 expression in CD45^+^F4/80^+^ cells with (Aβ40^+^) or without (Aβ40^-^) Aβ40 engulfment at 12w of age. *N* = 3 in Tg-SwDI/B^+/+^ group, *N* = 3 in Tg-SwDI/B^+/-^ group, *N* = 4 in WT group. Data are presented as mean values ± SEM with the indicated significance (compared with WT by two-tailed Student’s *t* test). Source data are provided as a Source Data file.

Our data illustrate that the increment of Aβ40 in CAA patients (Fig. S4a) was positively correlated with the extra migrasome production by macrophage lineage cells (Fig. 1g). Considering the crucial role of Aβ40 in CAA pathophysiology^3,4^, we evaluated the impact of Aβ40 on migrasome productivity of macrophage lineage cells. Human peripheral blood monocytes (Fig. S4b) and monocyte-derived macrophages were treated with plasma of CAA patients. With immunostaining, we found that CAA plasma elicited retraction fibers which were attached with TSPAN4-expressing oval membrane bound vesicles, conforming to the reported migrasome structure^10^ (Fig. 2a). At the meantime, TSPAN4 expression in monocytes and macrophages increased upon CAA plasma stimulation (Fig. 2b). Ablation of Aβ40 with monoclonal antibodies (Fig. S4c) abolished the migrasome-inducing effects of CAA plasma (Fig. 2a, b). Moreover, treatment of Aβ40 promoted migrasome production in human monocytes and macrophages (Fig. S4d), indicating that Aβ40 was a sufficient and necessary inducer of macrophage-derived migrasome.

Three subsets of macrophage lineage cells in brain, namely monocyte derived macrophage, brain resident PVM and microglia, contribute to Aβ40 clearance^13^. In vitro, mice bone marrow derived macrophages (BMDM) displayed active migrasome production in response to Aβ40 stimulation (Fig. S5a, b) in time (Fig. S5c) and dose (Fig. S5d) dependent manner. The migrasome inducing effect of Aβ40 was also evident in microglia (Fig. S5e, f). In the brain of Tg-SwDI/B and 5xFAD mice, Iba1^+^ microglia/macrophages (Figs. 2c and S6), CD206^+^ PVM (Fig. S7a) and F4/80^+^ infiltrated macrophages (Fig. S7b) with Aβ40 engulfment displayed active production of migrasomes (TSPAN4^+^ and/or TSPAN9^+^). To trace the origin of excessive migrasomes among macrophage lineage cells, microglia, PVM or circulating monocytes were depleted respectively in Tg-SwDI/B mice. With reliable depletion efficiency (Fig. S7c–e), we found that elimination of any macrophage subset down-regulated migrasome quantity in CAA brains as assessed with flow cytometry (Fig. S7f), immunostaining (Fig. S7g) and TEM (Fig. S7h). We thus infer that all brain macrophage lineage cells that confront Aβ40, including microglia, PVM and monocyte-derived infiltrated macrophages, contribute to the excessive migrasome production in CAA.

We further explored the chronological order of Aβ40 accumulation, Aβ40 engulfment by macrophages and migrasome production with heterozygous and homozygous Tg-SwDI/B mice (Fig. 2d). As assessed with ELISA, we found that plasma Aβ40 concentration in homozygous Tg-SwDI/B mice was elevated at 8w (Fig. 2di). Increment of Aβ40 containing macrophages (CD45^+^F4/80^+^Aβ40^+^) in peripheral blood was recorded at 12w of age (Fig. 2dii). Accordingly, TSPAN4 expression in macrophages (CD45^+^F4/80^+^TSPAN4^+^) in the peripheral blood was up-regulated at 12w, synchronizing with their phagocytosis of Aβ40 (Fig. 2diii). In heterozygous Tg-SwDI/B mice with reduced Aβ40 production, elevation of plasma Aβ40 was delayed to 12w (Fig. 2di). Aβ40 engulfment (Aβ40^+^) (Fig. 2dii) and TSPAN4 expression (TSPAN4^+^) (Fig. 2diii) of macrophages (CD45^+^F4/80^+^) were comparable to that of WT until 12w of age. Notably, TSPAN4 expression was higher in macrophages that had engulfed Aβ40 (Aβ40^+^) than those without Aβ40 phagocytosis (Aβ40^-^) in WT, heterozygous and homozygous Tg-SwDI/B mice (Fig. 2e). In vitro, BMDM was treated with cytochalasin D (CyD) to inhibit phagocytosis before Aβ40 stimulation. We recorded that the TSPAN4-inducing and migrasome-eliciting effect of Aβ40 was abolished when without being engulfed by BMDM (Fig. S8a, b). Knock-down of TSPAN4 (TSPAN4 KD) in BMDM down-regulated the migrasome productivity (Fig. S8d, e) although Aβ40 engulfment was un-affected (Fig. S8f). In contrast, TSPAN4 over expression led to excessive migrasome production (Fig. S8e). These results illustrate that the migrasome-inducing effect of Aβ40 relies on its internalization by macrophages and the subsequent increased TSPAN4 expression. We thus draw the conclusion that accumulation of Aβ40 triggers its engulfment by macrophage lineage cells, which subsequently induces TSPAN4 expression and migrasome production.

To be noticed, oligomerized Aβ40 displayed comparable migrasome-inducing effects as Aβ40 monomer (Aβ40 used through the study) (Fig. S8g, h). However, other amyloid protein, including Aβ1-42 (Aβ42), an analogue of Aβ40 which attributes to Alzheimer’s disease (AD), could not up-regulate migrasome production or TSPAN4 expression in human monocytes, monocyte-derived macrophages (Figs. 2a, b and S4e), or mouse BMDM in either monomer or oligomer (Fig. S8g, h). Aβ1-16 (Aβ16) and Aβ22-35 could neither induce significant migrasome release (Fig. S8g, h), emphasizing the specificity of Aβ40 in migrasome induction in macrophage lineage cells. Cerebral micro bleeding (CMB) is a common neuroimaging symptom in CAA. To evaluate the impact of cerebral bleeding on migrasome production, mouse BMDM was treated with red blood cells (RBC) (macrophage: RBC = 1: 5) for 6 h. Migrasome production of BMDM remained stable after RBC stimulation (Fig. S8g, h), indicating the innocence of cerebral bleeding in the excessive migrasome production. On the other hand, we recorded that several pro-inflammatory mediators, including LPS, TNFα, IFNγ, IL-1β and IL-17A, slightly upregulated TSPAN4 expression in BMDM but failed to elicit extra migrasome production (Fig. S8g, h). The data illustrate that among the macrophage-confronted stimulators in CAA, Aβ40 is a fundamental inducer of extra migrasomes.

### Migrasomes derived from Aβ40-stimulated macrophages are destructive to BBB

**Fig. 3:**
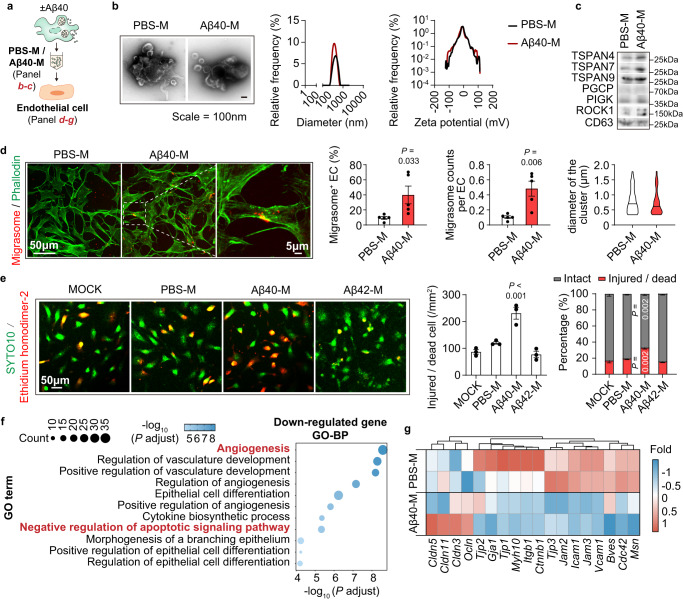
Migrasomes derived from Aβ40-stimulated macrophages are injurious to blood vessel endothelial cells. **a** Diagram of migrasome collection and treatment to endothelial cells. **b** Left: representative TEM images of the isolated migrasomes after negative staining. Middle: diameter of PBS-M and Aβ40-M. Right: Zeta potential of the isolated migrasomes. Data are representative of 3 biologically independent experiments. Lines in plots represent mean values. **c** Expression of migrasome markers in the purified migrasomes was assessed with western blot. Data are representative of 3 biologically independent experiments. **d**–**g** Migrasomes were treated to endothelial cells at a concentration of 50 μg/ml for 2 h. **d** Frequency of endothelial cells (green) attached with migrasomes (red) and the number of migrasomes bond to each endothelial cell were calculated. Data are representative of 5 biologically independent experiments. Diameter of cluster for PBS-M (*N* = 201) and Aβ40-M (*N* = 160) was also quantified. Data are presented as mean values ± SEM with the indicated significance (by two-tailed Student’s *t* test). **e** Endothelial cells were treated with PBS-M, Aβ40-M or Aβ42-M then subjected to SYTO10 and Ethidium homodimer-2 staining. The number of intact cells (SYTO10^+^Ethidium homodimer-2^-^) and injured/dead cells (SYTO10^+^Ethidium homodimer-2^+^) was calculated. Data are representative of 3 biologically independent experiments. Left: representative images showing the cytotoxic property. Middle: Comparison of injured/dead cells in endothelial cells treated with PBS-M, Aβ40-M or Aβ42-M. Right: Percentage of intact cells (grey bars) and injured/dead cells (red bars) in PBS-M-, Aβ40-M- or Aβ42-M-treated endothelial cells. Data are presented as mean values ± SEM with the indicated significance (compared with MOCK by one-way ANOVA followed by Tukey’s post-test). **f**–**g** Endothelial cells were treated with 50 μg/ml of PBS-M or Aβ40-M for 2 h then subjected to bulk RNAseq. *N* = 2 in each group. **f** Gene Ontology-biological process (GO-BP) enrichment of the downregulated genes in Aβ40-M treated endothelial cells. **g** Heatmap showing the transcription of tight junction protein related genes of PBS-M and Aβ40-M treated endothelial cells. Source data are provided as a Source Data file.

**Fig. 4:**
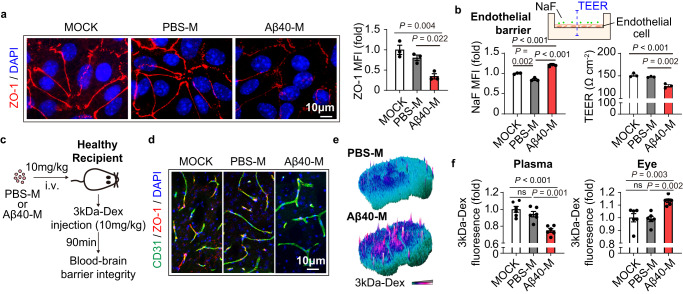
Migrasomes derived from macrophages upon Aβ40 stimulation directly cause brain blood vessel damage. **a** Primary brain blood vessel endothelial cells were treated with 50 μg/ml of PBS-M or Aβ40-M derived from BMDM for 2 h. ZO-1 expression (red) in migrasome-treated endothelial cells was assessed with immunostaining and ZO-1 MFI was calculated. Data shown are representative of 3 biologically independent experiments. Data are presented as mean values ± SEM with the indicated significance (by one-way ANOVA followed by Tukey’s post-test). **b** Endothelial cells were seeded on top of a cell culture insert (pore diameter = 0.4 μm) as a simulation of endothelial barrier. Permeability to Fluorescein sodium (NaF) and the trans-endothelial electrical resistance (TEER) of the barrier were assessed after 2 h of migrasome treatment (50 μg/ml). Data shown are representative of 3 biologically independent experiments. Data are presented as mean values ± SEM with the indicated significance (by one-way ANOVA followed by Tukey’s post-test). **c**–**f** Healthy C57/BL6 WT mice (age = 8–10w) were treated with 4 serial doses of Raw264.7 cell derived migrasomes (10 mg/kg, i.v. injected at day 1, 2, 3, 5). Six days after the first treatment (day 7), recipients were injected with 10 mg/kg 3kDa-Dextran at 90 min before sacrifice. Mice were perfused with PBS then 4% paraformaldehyde after peripheral blood collection. **c** Diagram indicating the experimental time line. **d** Coronal brain sections of the recipients were collected and subjected to immunostaining of CD31 (green) and ZO-1 (red) to evaluate the tight junction integrity. **e** Leakage of 3kDa-Dextran in the brain parenchyma was assessed with fluorescent microscopy. **f** Fluorescent intensity of 3kDa-Dextran in the plasma and eyeballs of the recipients were assessed with a 96-well plate fluorescence reader. *N* = 6 in each group. Data are presented as mean values ± SEM with the indicated significance (by one-way ANOVA followed by Tukey’s post-test). Source data are provided as a Source Data file.

Since TSPAN4 expression of macrophages was positively associated with plasma NeFL level (Fig. S2d), we inferred that macrophage-derived migrasomes contributed to BBB disruption and brain injury in CAA. To test the hypothesis, migrasomes derived from Aβ40-stimulated BMDM were isolated and the destructive property was analyzed (Fig. 3a). The purified vesicles displayed typical migrasome structure as revealed by TEM (Fig. 3b, left), homogeneous diameter analysis (Fig. 3b, middle), structural stability analysis (Fig. 3b, right) and immunostaining (Figs. S9a and S10b left). Expression of migrasome markers in the isolated vesicles was confirmed with western blot (Fig. 3c). Compared with migrasomes derived from unstimulated BMDM (PBS treated, designated as PBS-migrasomes, PBS-M), those produced by Aβ40-treated BMDM (designated as Aβ40-migrasomes, Aβ40-M) showed strong adherence to endothelial cells (Fig. 3d). Aβ40-M caused endothelial injury as soon as 2 h as assessed by SYTO10/Ethidium homodimer-2 staining (Fig. 3e), flow cytometric analysis of Annexin V/PI (Fig. S9c) and LDH release (Fig. S9d). Several caspase signaling pathways were activated in Aβ40-M treated endothelial cells, including Caspase-3, 7, 8, 9 (Fig. S9e), which further revealed the cytotoxic property of Aβ40-M. Consistently, Aβ40-M were injurious to other BBB components, including astrocytes (Fig. S9f) and pericytes (Fig. S9g). Bulk RNA sequencing (RNAseq) and the subsequent Gene Ontology-biological process (GO-BP) analysis revealed that genes associated with survival (*GO: Negative regulation of apoptotic signaling pathway*) and proliferation (*GO: Angiogenesis*) were down-regulated in Aβ40-M treated endothelial cells (Fig. 3f). Moreover, transcription of multiple tight junction associated genes was declined, including the zona occludens-1 (ZO-1) encoding gene *Tjp1* (Fig. 3g). Accordingly, ZO-1 protein level and its continuity in endothelium interval were decreased after Aβ40-M treatment as assessed with immunostaining (Fig. 4a). To evaluate the endothelial barrier integrity upon migrasome stimulation, brain blood vessel endothelial cells were seeded on a cell culture insert (pore diameter = 0.4 μm). Permeability to Fluorescein sodium (NaF) and the trans-endothelial electrical resistance (TEER) of the barrier were assessed at 2 h after migrasome administration (Fig. 4b). We recorded increased NaF-fluorescence intensity in the lower chamber and reduced TEER in Aβ40-M treated endothelial barrier (Fig. 4b). To confirm the BBB damaging function of migrasomes, BMDM were first pre-treated with Aβ40 then cocultured with brain blood vessel endothelial cells in a transwell based system. We found that endothelial cells cultured with the Aβ40-stimulated BMDM had down-regulated expression of the tight junction protein ZO-1 (Fig. S10a). Inhibition of migrasome production by knocking down TSPAN4 expression in BMDM efficiently ameliorated the injurious impacts of macrophages, while addition of BMDM-derived Aβ40-M in the coculture system reversed the protection of TSPAN4 KD (Fig. S10a). Notably, we found that migrasome construction was indispensable for their destructive effects. When the intrinsic structure was destroyed with ultrasound, Aβ40-M was no longer cytotoxic to endothelial cells (Fig. S9h) nor damaging to endothelial barrier (Fig. S9i, j). Compared with Aβ40-M, migrasomes derived from Aβ42-treated BMDM (Aβ42-M) displayed similar adhesiveness (Fig. S9b) but turned out to be harmless to endothelial cells (Figs. 3e and S9c, d).

We next examined the pathogenicity of migrasomes derived from Aβ40-stimulated macrophages. Migrasomes produced by Aβ40- or PBS-treated Raw264.7 cells (mouse macrophage cell line), i.e. Aβ40-M or PBS-M (Fig. S10b–f), were transferred to wild type healthy C57/BL6 mice (10 mg/kg, i.v., injected at day 1, 2, 3, 5). At day 7, each recipient was injected with a single dose of 3kDa-Dextran (3kDa-Dex, 10 mg/kg, i.v.) at 90 min before sacrifice (Fig. 4c). Attachment of Aβ40-M to brain blood vasculature was confirmed with fluorescent microscopy (Figs. S10g and S14f). Consistent with our in vitro data, administration of Aβ40-M caused BBB injury in vivo. Brain microvessels of the recipients displayed decreased ZO-1 expression (Fig. 4d). Leakage of 3kDa-Dextran into brain parenchyma (Fig. 4e) and eyeballs (Fig. 4f) from peripheral blood (Figs. 4f and S10h) was evident in the recipients. Meanwhile, plasma NeFL was elevated in Aβ40-M transferred mice, which further revealed the neuroaxonal degeneration caused by the migrasomes (Fig. S10i). Our data demonstrate that migrasomes derived from Aβ40-stimulated macrophages are destructive to BBB, and may initiate the self-enforcing pathophysiology of Aβ deposition and brain injury.

### Macrophage-derived migrasomes impair BBB through complement dependent cytotoxicity (CDC) in CAA

**Fig. 5:**
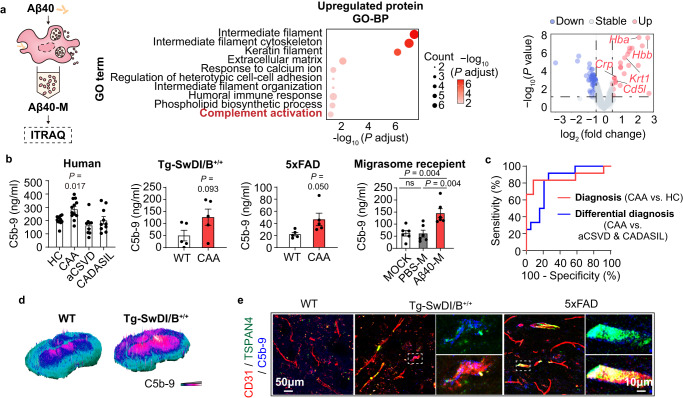
Migrasomes derived from Aβ40 stimulated macrophages facilitate complement dependent cytotoxicity to brain blood vessels. **a** BMDM derived migrasomes were collected and subjected to ITRAQ. *N* = 3 in both groups. Left: Diagram indicating the experimental work flow. Middle: Upregulated proteins in Aβ40-M compared with PBS-M were projected to GO-BP analysis. The top 10 GO terms were listed. Right: Volcano plot showing the deferentially expressed proteins in Aβ40-M compared with PBS-M. Gene names of proteins enriched in the GO term *Complement activation* were emphasized. **b** C5b-9 concentration in HCs (*N* = 12), CAA patients (*N* = 12), aCSVD patients (*N* = 9) and CADASIL patients (*N* = 10) (left), CAA mice models (*N* = 5 in Tg-SwDI/B^+/+^ mice and *N* = 5 in 5xFAD mice) and WT (*N* = 5 representively in independent experiments when compared to Tg-SwDI/B^+/+^ and 5xFAD mice) (middle), as well as migrasome recipients (*N* = 6 in each group, right). Data are presented as mean values ± SEM with the indicated significance (by one-way ANOVA followed by Tukey’s post-test or two-tailed Student’s *t* test). **c** Differential efficacy for CAA (TRUE) from HC (FALSE, diagnosis) or from patients with aCSVD or CADASIL (FALSE, differential diagnosis) was estimated with ROC analysis. *N* = 12 in CAA groups. *N* = 12 in HC group. *N* = 9 in aCSVD group. *N* = 10 in CADASIL group. **d**, **e** CAA models (Tg-SwDI/B^+/+^ mice) and WT C57/BL6 mice were sacrificed at 24w of age. Coronal brain sections were collected and subjected to immunostaining. **d** C5b-9 deposition in brain parenchyma. **e** Immunostaining of CD31 (red), TSPAN4 (green) and C5b-9 (blue). Data shown are representative of 4 biologically independent experiments. Source data are provided as a Source Data file.

To explore the mechanisms of migrasome-induced BBB damage, Aβ40-M and PBS-M were subjected to isobaric tags for relative and absolute quantification (ITRAQ) (Fig. 5a). GO-BP analysis of the proteins identified in PBS-M revealed that the function of macrophage-derived migrasomes was associated with regulation of phagocytosis and metabolism in physiological context (Fig. S11a). Nevertheless, upon Aβ40 stimulation, the physiological effect of migrasomes seemed to be diminished as multiple metabolism associated proteins were no longer enriched in Aβ40-M (Fig. S11b). Meanwhile, the GO term of *Cell adhesion molecule binding* was specifically enriched with proteins in Aβ40-M (Fig. S11b). Aβ40-M contained abundant adhesion/cell-cell contact related proteins including fibrinogen alpha chain (FGA) and apolipoprotein A-I (APOA1) (Fig. S11c). Of interest, multiple mitochondrial proteins were identified in both PBS-M and Aβ40-M with comparable quantity, indicating that mitochondrial components were packed in macrophage-derived migrasomes as those derived from neutrophils^11^.

Several complement activation related molecules were found among the up-regulated proteins in Aβ40-M, namely CD5L, C reactive protein (CRP), keratin 1 (KRT1), hemoglobin A (HBA) and hemoglobin B (HBB) (Fig. 5a). Concentration of complement 5b-9 (C5b-9), the membrane attack complex (MAC) formed during complement activation, was increased in plasma of CAA patients (Fig. 5b) and showed favorable efficiency in distinguishment of CAA from aCSVD/CADASIL or HC (Fig. 5c). In consistence, we recorded elevated plasma C5b-9 level in Tg-SwDI/B, 5xFAD mice as well as the WT recipients of Aβ40-M (Fig. 5b). With immunostaining, we detected C5b-9 deposition in brains of CAA models (Fig. 5d, e) and Aβ40-M recipients (Fig. S12f), which co-localized with the migrasome marker TSPAN4 along brain blood vessels (CD31^+^). As revealed by Spearman correlation analysis, plasma C5b-9 level was positively associated with TSPAN4 expression of monocytes and plasma NeFL concentration in CAA (Fig. S12a–e), suggesting the association among complement activation, macrophage-derived migrasomes and brain injury. With in vitro experiments, we found that the damaging effects of Aβ40-M to endothelial cells was abolished when complements in FBS were deactivated, while replenishment with complement mixture in the culture system re-produced the injury (Fig. S13a, b). Therefore, we propose that macrophage-derived migrasomes damage BBB through activating CDC in CAA.

### CD5L is docked to blood vessel wall by macrophage-derived migrasomes to facilitate CDC in CAA

We next sought to uncover the molecular mechanism of migrasome-mediated CDC. According to the ITRAQ data, both PBS-M and Aβ40-M contained complement isoforms such as C1qa, C1qc, C1ra, C3 and C4b. Nevertheless, the level of the complement proteins was comparable between PBS-M and Aβ40-M. Therefore, we infer that the injurious effect of Aβ40-M was independent of the intra-migrasome complement components. In validation of the ITRAQ result, Aβ40 stimulation increased CD5L, HBA, HBB and CRP expression in both BMDM (Fig. S14a) and Aβ40-M (Fig. S14b). Notably, increment of CD5L was the most prominent (Fig. S14b). CD5L, with the alias of apoptosis inhibitor of macrophage (AIM), is mainly produced by macrophages in inflamed tissue, which promotes macrophage survival and exacerbates inflammatory reactions^14^. Package of CD5L into migrasomes seemed to be dependent on Aβ40 internalization and TSPAN4-mediated migrasome production in macrophages. Inhibition of Aβ40 phagocytosis by CyD (Fig. S8c) or knocking down TSPAN4 (Fig. S8d) not only down-regulated migrasome production but also decreased CD5L concentration in the extracellular vesicles (Fig. S8c, d).

To evaluate the pro-CDC effect of CD5L, the in vitro endothelial barrier model was treated with CD5L at various concentration with or without complement mixture (Fig. S15a). We found that CD5L caused endothelial injury with concentration of over 100 ng/ml and the presence of complement mixture (Fig. S15a) through CDC (Fig. S15b, c). To confirm the necessity of CD5L in the migrasome-associated BBB damage, Raw264.7 cells were stimulated with Aβ40 then cocultured with brain blood vessel endothelial cells in a transwell based system. We found that co-culture of Aβ40-stimulated WT Raw264.7 cells with brain blood vessel endothelial cells resulted in ZO-1 loss and complement activation (C5b-9^+^) (Fig. S14c), while knockout of CD5L inhibited the damage, which was reversed after addition of CD5L (Fig. S14c). The result indicated that CD5L mediated the BBB damage caused by macrophage derived migrasomes. Mechanistically, the injurious effect of CD5L was independent of its established receptor CD36^15^ (Fig. S15d). Consistent with previous report^16^, we found that CD5L decreased the expression of CD59 which was a complement inhibitory molecule (Fig. S15e). Therefore, we demonstrated that CD5L facilitated CDC in brain blood vessel in CAA through activating complement and weakening the resistance to CDC.

**Fig. 6:**
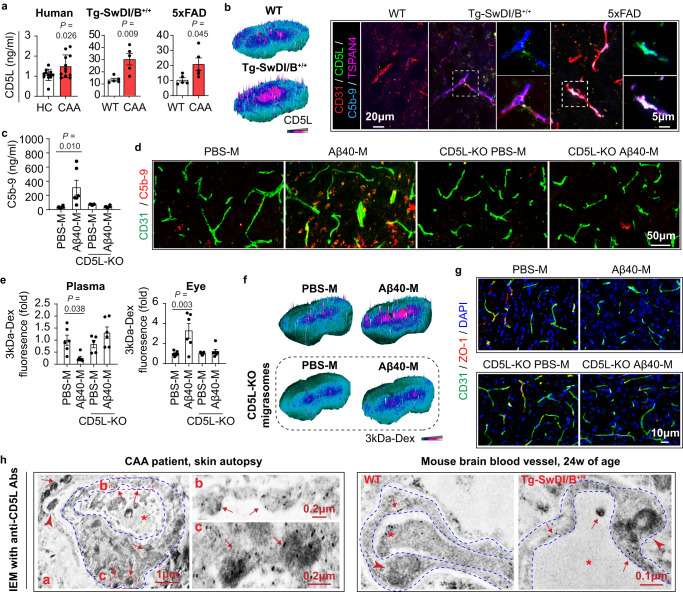
CD5L is docked to brain blood vessels by macrophage-derived migrasomes upon Aβ40 stimulation and is indispensable for migrasome-mediated BBB damage. **a** Plasma CD5L concentration of CAA patients (*N* = 12) and HC (*N* = 12) as well as CAA models (*N* = 5 in each group) and WT C57/BL6 mice (*N* = 5). Data are presented as mean values ± SEM with the indicated significance (by two-tailed Student’s *t* test). **b** Left: CD5L deposition in mice brain was evaluated with immunostaining. Right: Immunostaining of CD31 (red), CD5L (green), C5b-9 (blue) and TSPAN4 (purple). Data are representative of 4 biologically independent experiments. **c** Plasma C5-9 concentration of mice. *N* = 6 in PBS-M, Aβ40-M and CD5L KO Aβ40-M transferred mice and *N* = 5 in CD5L KO PBS-M treated group. Data are presented as mean values ± SEM with the indicated significance (by one-way ANOVA followed by Tukey’s post-test). **d** C5b-9 deposition in brain as assessed with immunostaining. Data are representative of 3 biologically independent experiments. **e** Fluorescent intensity of 3kDa-Dextran in the plasma and eyeballs of the recipients. *N* = 6 in PBS-M, Aβ40-M and CD5L KO Aβ40-M transferred mice and *N* = 5 in CD5L KO PBS-M treated group. Data are presented as mean values ± SEM with the indicated significance (by one-way ANOVA followed by Tukey’s post-test). **f** Leakage of 3kDa-Dextran in brain parenchyma was assessed with fluorescent microscopy. Data are representative of 3 biologically independent experiments. **g** Coronal brain sections of the recipients were subjected to immunostaining of CD31 (green) and ZO-1 (red). Data are representative of 3 biologically independent experiments. **h** Skin autopsy sample of a CAA patient (Left) and brain tissue of WT and Tg-SwDI/B^+/+^ mice (24w of age) (Right) were subjected to IEM with CD5L staining. Blue dash lines outline micro-vessel wall. Red stars emphasize blood vessel lumen. Red arrow heads emphasize peri-vessel macrophages. Red arrows emphasize CD5L deposition in blood vessels within migrasome-like structures. Data are representative of 3 biologically independent experiments. Source data are provided as a Source Data file.

We further study the role of CD5L in migrasome-mediated CDC in CAA. Increased plasma CD5L was recorded in both CAA patients and murine models (Fig. 6a). CD5L deposition along brain blood vessels (CD31^+^) in close proximity with migrasomes (TSPAN4^+^) and MAC (C5b-9^+^) was observed in Tg-SwDI/B and 5xFAD mice (Fig. 6b). CD5L deficiency in Aβ40-M abolished the CDC initiating (Figs. 6c, d,  S14e,  S15f) and BBB damaging effects (Figs. 6c–g, S14e, S15g) both in vivo and in vitro. Similarly, Aβ40-EVs derived from CyD-treated WT BMDM (Fig. S8c) or TSPAN4 KD BMDM (Fig. S8d) which contained little CD5L failed to induce MAC formation (Fig. S15h, j) and endothelial injury (Fig. S15i, k). These data indicating that CD5L was indispensable in the complement-dependent BBB injury caused by macrophage-derived migrasomes in CAA.

Interestingly, we found that the pro-CDC effect of CD5L relied on their enrichment in blood vessels by migrasomes. Immuno-electron microscopy (IEM) showed that CD5L was docked to blood vessel wall by migrasomes in CAA patient and mouse models (Fig. 6h), which elevated local CD5L concentration to a damaging level (~100 ng/ml, estimated according to Fig. 1a). Confocal microscopy revealed that transfer of Aβ40-M increased the local CD5L level in blood vessels, which was abolished when CD5L was knocked out in the source Raw264.7 cells (Fig. S14f). When intrinsic structure of migrasomes was destroyed with ultrasound that undermined their adherence to endothelial cells (Fig. S15l), Aβ40-M failed to enrich CD5L on endothelial barrier (Fig. S15l) or induce CDC (Fig. S9h–j) albeit the CD5L concentration remained stable (Fig. S15m). Therefore, we conclude that Aβ40-induced macrophage-derived migrasomes dock CD5L on blood vessels thus facilitating CDC in BBB.

### Complement inhibitory therapy ameliorates migrasome-mediated BBB impairment in CAA

**Fig. 7:**
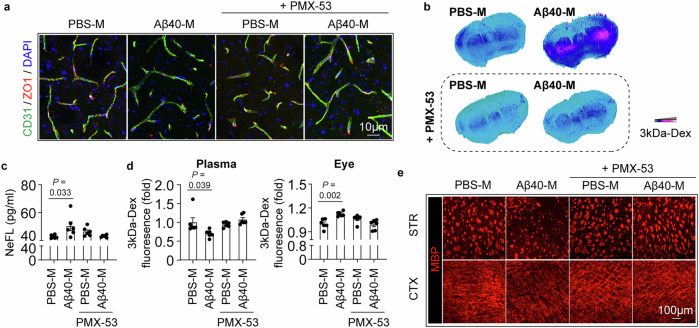
Complement inhibitory treatment protects against migrasome mediated BBB injury. Healthy C57/BL6 WT mice (age = 8-10w) were treated with 4 serial doses of Raw264.7 cell derived migrasomes (10 mg/kg, i.v. injected at day 1, 2, 3, 5) in addition with PMX-53 treatment (0.25 mg per mouse, o.p.). Six days after the first migrasome injection (day 7), recipients were injected with 10 mg/kg 3kDa-Dextran at 90 min before sacrifice. Mice were perfused with PBS then 4% paraformaldehyde after peripheral blood collection. *N* = 6 in each group. **a** Coronal brain sections of the recipients were collected and subjected to immunostaining of CD31 (green) and ZO-1 (red) to evaluate the tight junction integrity. **b** Leakage of 3kDa-Dextran in the brain parenchyma was assessed with fluorescent microscopy. **c** Plasma NeFL concentration of the recipients. *N* = 6 mice in each group. Data are presented as mean values ± SEM with the indicated significance (compared with PBS-M transferred group by one-way ANOVA followed by Tukey’s post-test). **d** Fluorescent intensity of 3kDa-Dextran in the plasma and eye balls of the recipients. *N* = 6 mice in each group. Data are presented as mean values ± SEM with the indicated significance (compared with PBS-M transferred group by one-way ANOVA followed by Tukey’s post-test). **e** White matter integrity of recipients was assessed with MBP immunostaining. MBP MFI in striatum (STR) and cortex (CTX) was calculated. *N* = 6 mice in each group. Statistics outcomes were presented in Fig. S16d. Source data are provided as a Source Data file.

**Fig. 8:**
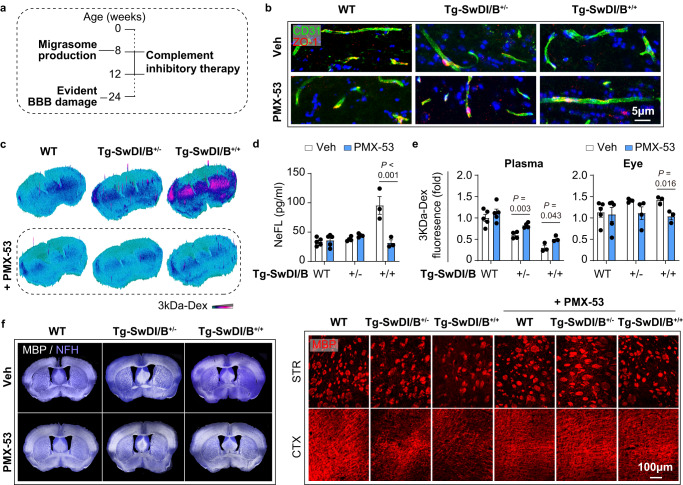
Complement inhibitory treatment ameliorates brain blood vessels damage in CAA. Tg-SwDI/B^+/+^, Tg-SwDI/B^+/-^ and WT mice were treated with complement inhibitory therapy, namely PMX-53 (0.25 mg per mouse, o.p.). Mice were sacrificed at 12w of age. *N* = 5 in each WT ± PMX-53 groups, *N* = 4 in Tg-SwDI/B^+/-^ ± PMX-53 groups; *N* = 3 in Tg-SwDI/B^+/+^ ± PMX-53 groups. **a** Major pathophysiological events (left) and time line of complement inhibition therapy (right) were displayed. **b** Coronal brain sections of mice were collected and subjected to immunostaining of CD31 (green) and ZO-1 (red) to evaluate the tight junction integrity. **c** Leakage of 3kDa-Dextran in the brain parenchyma was assessed with fluorescent microscopy. **d** Plasma NeFL concentration as assessed with ELISA. *N* = 5 mice in each WT ± PMX-53 groups, *N* = 4 mice in Tg-SwDI/B^+/-^ ± PMX-53 groups; *N* = 3 mice in Tg-SwDI/B^+/+^ ± PMX-53 groups. Data are presented as mean values ± SEM with the indicated significance (compared with vehicle treated group (Veh, water) by two-tailed Student’s *t* test. **e** Fluorescent intensity of 3kDa-Dextran in the plasma and eyeballs was assessed with a 96-well plate fluorescence reader. *N* = 5 mice in each WT ± PMX-53 groups, *N* = 4 mice in Tg-SwDI/B^+/-^ ± PMX-53 groups; *N* = 3 mice in Tg-SwDI/B^+/+^ ± PMX-53 groups. Data are presented as mean values ± SEM with the indicated significance (compared with vehicle treated group by two-tailed Student’s *t* test). **f** White matter integrity was assessed with MBP/NFH immunostaining. MBP MFI in striatum (STR) and cortex (CTX) was evaluated. Source data are provided as a Source Data file.

Complement activation is the terminal step of the pathological chain which links BBB damage with deposition of macrophage-derived migrasomes in CAA. Therefore, we infer that complement activation is a therapeutic target against migrasome-mediated BBB damage. With the in vitro endothelial barrier model, we found that complement inhibitory treatments, including PMX-53 (C5a receptor antagonist), Avacopan (C5a receptor antagonist) and Eculizumab (monoclonal antibody against C5), efficiently abolished the BBB-damaging effect of Aβ40-M (Fig. S16a, b). Considering the practicability, the orally active PMX-53 was used in the subsequent study. We found that PMX-53 reversed the injurious effects of Aβ40-M in vivo. The WT Aβ40-M recipients treated with PMX-53 displayed down-regulated plasma C5b-9 (Fig. S16c), preserved BBB integrity (Fig. 7a–d) and ameliorated white matter loss (Fig. 7e, Fig. S16d). To evaluate the therapeutic potential of complement inhibitory treatment in CAA, Tg-SwDI/B mice were treated with PMX-53 from 8w of age. Mice were sacrificed at 12w when BBB impairment was recordable according to previous report^17^ (Fig. 8a). We documented that PMX-53 resolved complement activation (Fig. S17a), ameliorated BBB injury (Fig. 8b–e) and improved white matter integrity (Fig. 8f) in the CAA models without altering plasma CD5L level (Fig. S17b). Unexpectedly, the concentration of plasma Aβ40 was down-regulated by PMX-53 in Tg-SwDI/B mice (Fig. S17c) probably through breaking out the vicious circle of Aβ40 deposition and blood vessel injury. These data support that complement inhibition represents a promising strategy in CAA therapy.

## Discussion

The current study reveals the implication of macrophage-derived migrasomes in CAA progression. We show that macrophages pack disease promoting molecules in migrasomes including CD5L, which facilitates complement activation and BBB injury. We propose that complement activation is of diagnostic significance and a potential therapeutic target in CAA.

Migrasomes are extracellular vesicles that are produced during cellular migration^10,18^. In different physiological contexts, migrasomes are endowed with diverse responsibilities^19^. Neutrophil represents an important cellular source of migrasomes. Neutrophil-derived migrasomes mediate mitochondrial iteration which maintains mitochondrial quality and cell viability^11^. In the current study, we record that migrasomes production increases in CAA. However, migrasome productivity in neutrophils is comparable between CAA patients and healthy controls. Unexpectedly, it is macrophage lineage cells that are responsible for the excessive migrasome production. Similar to neutrophil-derived migrasomes^11^, migrasomes produced by macrophages contain mitochondrial components. Nevertheless, the level of mitochondrial proteins is comparable between PBS-M and Aβ40-M, which indicates the innocence of mitochondrial components to the pathogenic impacts of migrasomes.

Our results illustrate that Aβ40 is the fundamental inducer of macrophage-derived migrasomes. In Tg-SwDI/B^+/+^ mice, increment of Aβ40 in the circulation is first evident at 8w of age. Aβ40 overload and migrasome production of macrophages take the stage simultaneously at 12w. Macrophages with Aβ40 engulfment that display intense migrasome production rather than that without Aβ40 internalization. Treatment of CAA plasma with increased Aβ40 concentration elicits migrasome production in human monocytes and macrophages while the effect is abolished by Aβ40 ablation, which emphasizes the fundamental role of Aβ40 in migrasome induction. Interestingly, Aβ40 monomers and oligomers displayed similar migrasome-inducing effect, which is probably attributed to their similar property as danger associated molecular pattern (DAMP)^20^. Aβ40 has long been regarded as a potential bio-marker of CAA. Despite the inconsistent results of plasma Aβ40 level, it is in agreement that Aβ40 deposits and accumulates in micro-vessels of CAA patients and animal models. Therefore, the migrasome-inducing effect of Aβ40 is of generality in CAA.

Bio-markers for CAA diagnosis have been explored in the current study. In response to Aβ40 stimulation, multiple danger and stress associated molecules are packed in migrasomes by macrophages. CD5L is one of the up-regulated proteins in Aβ40-M which facilitates complement activation. Until now, the precise molecular mechanisms of protein sorting into migrasomes and migrasome production remain elusive. Besides, intra-cellular distribution of CD5L is largely unknown. How CD5L is sorted and transported to migrasome in macrophage lineage cells upon Aβ40 stimulation is to be studied. As far as we are concerned, since CD5L is a secretary soluble protein and could enhance macrophage phagocytosis^21^, it is possible that CD5L is first secreted upon Aβ40 stimulation, which was then engulfed together with Aβ40 into the phagosomes. The phagosomes containing indigestible Aβ40 oligomers and CD5L might be then transported into migrasomes. In regard of how CD5L inside migrasomes exerts their BBB damaging effects, we observed membrane fusion of migrasomes and endothelial cells, which facilitated the mutual contaction of CD5L and the migrasome recipient cells. Nevertheless, the molecular mechanisms mediating the interaction of migrasomes and recipients cells and the CDC initiating effect by CD5L remain to be study. The CDC enhancing effect of secreted CD5L has also been reported^16^ and we found that plasma CD5L level was increased in both CAA patients and animal models. However, in vitro experiments revealed that the concentration of CD5L in the plasma of CAA patients or animal models was not sufficient to induce CDC in endothelial cells. According to our data, the pro-CDC effect of CD5L inside the migrasomes relied on their enrichment in blood vessels through migrasome attachment. CD5L was docked to blood vessel wall by migrasomes, which elevated local CD5L concentration to a damaging level (~100 ng/ml). To be noted, the precise interacting proteins that mediate migrasome attachment to endothelial cells are still elusive. Upon Aβ40 stimulation, expression of CD5L in both macrophages and the macrophage-derived migrasomes was increased, indicating that Aβ40 induced CD5L production by macrophages and its packaging into migrasomes. We found that CD5L was indispensable for the BBB injuring property of Aβ40-induced macrophage-derived migrasomes. CD5L deficiency in the migrasomes abolished the CDC initiating and BBB damaging effects both in vivo and in vitro. Consequently, we infer that Aβ40 induced CD5L packaging to migrasome, resulting in BBB damage.

Increment of plasma CD5L is evident in patients with various disorders^22–25^, indicating that CD5L is released by macrophages under different injurious circumstances. We fail to identify the value of CD5L as a diagnostic marker of CAA. In contrast, C5b-9 displays impressive differential efficacy for CAA. Although complement deposition in brain tissue has been documented^26,27^, no concrete clues of CDC and its impacts on BBB integrity in CAA have been provided. Here, we prove that CDC contributes to blood vessel damage in CAA which is mediated by migrasome attachment. Moreover, complement inhibition protects against migrasome-mediated BBB injury in CAA.

Vascular dysfunction results in cognitive impairment in CAA. We recorded that migrasome productivity of leukocytes was associated with cognitive decline in CAA patients. However, migrasome count of the recruited AD patients maintained as that of HC. Comorbidity with CAA is common in AD patients especially at advanced stage of disease. To weaken the impact of CAA comorbidity, AD patients with neuroimaging manifestations of CAA were excluded from the current cohort. As a result, the recruited AD patients were mostly mile cases and their outcomes of MoCA and MMSE were below AD average^28^ and turned out to be comparable to that of CAA patients. Nevertheless, although the recruited AD patients did not show pronounced increment in circulatory migrasome count, the BBB damaging impacts of migrasomes in AD patients could not be neglected. On the other hand, we find that the migrasome count in CAA patients is positively correlated with their cognitive impairment, indicating that although CAA patients with moderate to severe cognitive dysfunction present elevated migrasome abundance in the circulation, those with mild cognitive impairment might not have increased migrasome production.

Conclusively, the current study elucidates the injurious property of macrophage-derived migrasomes in CAA. Migrasomes are excessively produced by macrophage lineage cells that encounter amyloid protein stimulation, which further facilitate complement dependent blood vessel damage. We propose that C5b-9 represents a potential biomarker for CAA and complement inhibitory treatment is of therapeutic potential. However, the biological and pathological roles of migrasomes in various contexts remain to be elusive. How CD5L is sorted/transported into migrasomes and the detailed molecular mechanisms mediating the interaction of CD5L, migrasomes and migrasome recipient cells are all interesting and important scientific questions that should be further investigated.

## Methods

### Study approval

The clinical studies were approved by the Medical Ethics Committee of the Third Affiliated Hospital of Sun Yat-Sen University. All participants had been given the informed consent according to the principles illustrated in Declaration of Helsinki. All Mice were used in accordance with the Guide for Care and Use of Laboratory Animals of the National Institute of Health, and protocols were approved by the Institutional Animal Care and Use Committee of The Third Affiliated Hospital of Sun Yat-sen University.

### Human material

Peripheral blood samples were provided by 12 healthy donors, 12 CAA patients, 28 aCSVD patients, 10 cerebral autosomal dominant arteriopathy with subcortical infarcts and leukoencephalopathy (CADASIL) patients, and 10 AD patients recruited in the Third Affiliated Hospital of Sun Yat-sen University from April 2021 to December 2021. Demographic characteristics of the research cohort are summarized in Table S1.

### Study population

A cohort consisting 12 patients with CAA, 28 patients with aCSVD, 10 patients with CADASIL, 10 patients with AIS, 10 patients with AD, and 12 healthy controls in neurology clinics in the Third Affiliated Hospital of Sun Yat-Sen University from July 2018 to December 2021 was recruited consecutively. Laboratory tests and standard MRI were performed in all recruited patients for cardiovascular risk factors screening and neuroimaging assessment respectively. All CAA patients recruited in the study were eligible for the inclusion criteria below^29^: (1) Age ≥ 55 y. (2) MRI or CT neuroimaging showing that multiple hemorrhages (ICH, CMB) restricted to lobar, cortical, or cortical-subcortical regions (cerebellar hemorrhage allowed), or single lobar, cortical, or cortical-subcortical hemorrhage and cortical superficial siderosis (focal or disseminated). (3) Absence of other cause of hemorrhage (differential diagnosis of lobar haemorrhages) including antecedent head trauma, hemorrhagic transformation of an ischemic stroke, arteriovenous malformation, haemorrhagic tumor, warfarin therapy with international normalization ratio > 3, vasculitis. To preclude the impacts of intracerebral haemorrhage on the results of blood sampling, patients with acute (with hemorrhagic stroke within 2 days) or sub-acute (2-30 days after hemorrhagic stroke)^30^ brain hemorrhage were excluded. Hemorrhagic stroke (chronic phase) was presented in 2 CAA patients in the recruited cohort (*N* = 12). The duration between last hemorrhagic stroke and blood sampling was 4 years and 10 years respectively. No evidence of hemorrhagic stroke was recorded in the remained 10 patients and only CMB and / or cSS were detected through MRI. All aCSVD patients recruited in the study were eligible for the inclusion criteria below: (1) With at least one arteriosclerotic risk factors including age > 55 y, smoking (≥ 10 cigarettes per day for at least 10 years), body mass index (BMI) > 28, hypertension, diabetes mellitus, impaired glucose tolerance (IGT), impaired fasting glucose (IFG), coronary heart disease, hyperlipidemia, hyperhomocysteinemia, symptomatic stroke history. (2) With at least one common CSVD symptoms including cognitive decline, gait and balance disturbance, parkinsonism, emotional or sleeping disorder, urinary and fecal dysfunction. (3) The cerebral small vascular pathology was defined as the presence of at least one of the following imaging markers: lacunaes (≥ 1 lesion), EPVS (rating scale ≥ 2), brain atrophy (GCA rating scale ≥ 1), deep WMH or periventricular WMH (defined as Fazekas ≥ 2), cSS (≥ 1 lesion), or CMB ( ≥ 1 lesion). MRI neuroimaging met the STandards for ReportIng Vascular changes on nEuroimaging (STRIVE) for CSVD^31^. Lacunes were defined as a round or ovoid subcortical cavity of cerebrospinal fluid signal between 3- to 15-mm in diameter on MRI; EPVS was defined as fluid-filled space following the course of penetrating vessel of cerebrospinal fluid signal on all the MRI sequences, which can be either linear (when parallel to the penetrating vessel), round or ovoid (when perpendicular to the penetrating vessel) with a diameter less than 3 mm, and the grading scale for EPVS is as follows: Grade 0 indicates no EPVS, Grade 1 indicates 1 to 10 EPVS, Grade 2 indicates 11 to 20 EPVS, Grade 3 indicates 21 to 40 EPVS, and Grade 4 indicates the presence of more than 40 EPVS^32^; Brain atrophy, which is not related to macroscopic injuries including trauma or stroke, was defined according to GCA (Global cortex atrophy) rating. The GCA scale assesses the extent of atrophy in the cortex and sulcal dilatation, with scores ranging from 0 to 3. The scoring system is as follows: Grade 0 indicates no cortical atrophy; Grade 1 indicates mild atrophy with some opening of sulci; Grade 2 indicates moderate atrophy with significant volume loss of gyri; and Grade 3 indicates severe atrophy characterized by “knife blade atrophy.”^33^; WMHs were defined as hyperintensity on T2-FLAIR in the white matter, and the severity of WMHs can be graded using the Fazekas scale: Fazekas grade 1 (mild): characterized by periventricular caps of the ventricles or punctate foci in the deep white matter; Fazekas grade 2 (moderate): characterized by a smooth periventricular halo or convergence of deep white matter lesions; Fazekas grade 3 (severe): characterized by confluent periventricular leukoaraiosis that extends into the subcortical deep white matter; cSS was defined as linear hypointensities along the cortical gyral surface; and CMBs were small hypointense lesions visible on susceptibility-weighted imaging (SWI) sequences, which were generally 2–5 mm in diameter, but sometimes up to 10 mm. Among the recruited aCSVD patients, 14 presented CMB and none showed evidence of cSS or hemorragic stroke. (4) No visible moderate-severe intracranial arteriosclerotic stenosis in MR angiography. (5) No ischemic stroke attributed to large cerebral arteries occlusion or cardiac embolism. Patients with other CSVD etiologies secondary to genetic inheritance, infection, autoimmune inflammation, neoplasm, trauma, toxication, radiation, metabolic cerebropathy, and sporadic cerebral amyloid angiopathy were excluded. Diagnoses of all CADASIL patients recruited in the study were confirmed by genetic testing in all cases, which is the gold standard for the diagnosis of CADASIL^34^. All AIS patients recruited in the study consecutively had an independently documented primary stroke event in combination with confirmed MRI evidence showing ischemic stroke. All AIS patients met the following inclusion criteria: (1) onset age ≥18 years. (2) first symptomatic ischemic stroke. (3) clinical evidence of motor, language, attention, visual, or memory deficits based on neurological examination. (4) time of enrolment: <7 days from stroke onset^35^. All AD patients recruited in the study were eligible for the inclusion criteria below: (1) Patients were classified as having a clinical phenotype if they exhibited an amnestic syndrome of the hippocampal type, or if their clinical presentation was consistent with one of the known atypical variants (posterior variant, logopenic variant of primary progressive aphasia, frontal variant). Preclinical or presymptomatic states of AD were defined as the absence of clinical symptoms of AD, whether typical or atypical. (2) With at least one of the following changes indicative of in-vivo Alzheimer’s pathology: a CSF profile consisting of decreased Aβ1–42 levels together with increased T-tau or P-tau concentrations, or an increased retention on amyloid tracer PET. (3) Absence of sudden onset or early occurrence of the following symptoms: gait disturbances, seizures, major and prevalent behavioural changes. (4) No clinical features including focal neurological features, early extrapyramidal signs, early hallucinations or cognitive fluctuation. To preclude the interference of vascular amyloid deposition to the impact of brain parenchymal amyloid deposition, AD patients with MRI or CT neuroimaging clues for CAA were precluded, which included multiple hemorrhages (ICH, CMB) restricted to lobar, cortical, or cortical-subcortical regions (cerebellar hemorrhage allowed), or single lobar, cortical, or cortical-subcortical hemorrhage and cortical superficial siderosis (focal or disseminated). Demographic and neuroimaging characteristics of the cohort are summarized in Tables S1 and S2.

### MRI protocol and neuroimaging assessment

MRI was performed on a GE 3.0-Tesla scanner MR750 (General Electric, Milwaukee, USA) with a standard eight-channel HRBRAIN coil. The MRI protocol included (i) axial T1 FLAIR (fluid-attenuated inversion recovery) weighted: repetition time (TR) = 1750ms, echo time (TE) = 24 ms, echo train length (ETL) = 10, bandwidth (BW) = 41.67 kHz, matrix = 320 × 224, filed of view (FOV) = 240 mm, slice thickness = 5 mm, spacing = 1, and number of excitations (NEX) = 1; (ii) axial T2-weighted FrFSE (fast recovery fast spin echo): TR = 5727 ms, TE = 93 ms, ETL = 32, BW = 83.3 kHz, matrix = 512 × 512, FOV = 240 mm, slice thickness = 5 mm, spacing = 1, and NEX = 1.5; (iii) T2 FLAIR weighted: TR = 8400 ms, TE = 145 ms, inversion time (TI) = 2100 ms, BW = 83.3 kHz, flip angle (FA) = 145°, matrix = 320 × 224, FOV = 240 mm, slice thickness = 5 mm, spacing = 1, and NEX = 1; (iv) axial three-dimensional time-of-flight MR angiography (3D-TOF MRA): TR = 25 ms, TE = 3.4 ms, FA = 20°, BW = 41.67 kHz, matrix size = 384 × 320, FOV = 200 mm, slice thickness = 0.8 mm, and NEX = 1; (v) Axial T2*-weighted angiography (SWAN): TR = 77.3 ms, TE = 45 ms, BW = 62.5 kHz, FA = 15°, matrix = 384 × 320, slice thickness = 1 mm, and NEX = 1. MRI DICOM (Digital Imaging and Communications in Medicine) data were analyzed by an experienced neuroradiologist (X.C.) in ORS Visual (Montreal, Quebec, Canada). Total aCSVD neuroimaging burden was assessed according to an ordinal CSVD score (0 to 4) based on CSVD imaging principal summarized in STRIVE recommendation^31^. One score was awarded when each of the following signs was presented: number of lacunae ≥ 1; number of CMBs ≥ 1; moderate to severe enlargement of BG-PVS; p-WMH Fazekas score 3 (extending into the deep white matter) or d-WMH Fazekas score 2 to 3 (early confluent or confluent). The CAA neuroimaging burden was assessed according to total MRI small vessel disease burden in cerebral amyloid angiopathy (0 to 6)^4^. We pre-specified an ordinal scale representing the total burden of small vessel disease in CAA by incorporating the four most characteristic MRI markers of the disease (lobar CMBs, cSS, CSO-PVS and WMH). For lobar CMBs, a point was awarded if 2-4 CMBs were present and two points for ≥ 5 CMBs. Presence of cSS was awarded with one point if focal and two points if disseminated. Presence of CSO-PVS was counted if there were moderate-to-severe (grade 3-4, i.e. >20) PVS (one point if present). Presence of WMH was defined as either (early) confluent deep (i.e. the region between juxtacortical and ventricular areas) WMH (Fazekas score ≥ 2) or irregular periventricular WMH extending into the deep white matter (Fazekas score 3) (one point if either present).

### Animals

Wild-type mice (WT, C57/BL6, purchased from GemPharmatech, n000013), Tg-SwDI/B mice (purchased from the Jackson Laboratory, 007027) and 5xFAD mice (purchased from Aniphe Biolaboratory Inc, ALF1112015) were reproduced in the animal facility of Sun Yat-sen university (Animal using license: SYXK 2007-0081). Tg-SwDI/B mice are CAA models with C57/BL6 background, which are generated with knocked in of human amyloid-β precursor protein (*APP*) gene with three familial mutations and develop CAA at ~24w of age, were utilized as CAA murine models. 5xFAD mice have five FAD mutations under the promoter of Thy1: APP K670N/M671L (Swedish) I716V + (Florida) + V717I (London) and PS1 M146L + L286V (5xFAD). In migrasome transfer experiments, macrophage-derived migrasomes were transferred intravenously into healthy C57/BL6 WT mice every 2 days. Recipients were sacrificed at day 7 of experiment. In experiments of with complement inhibitory treatment, the synthetic peptide PMX-53, an orally active C5a receptor antagonist, was administered to mice (0.25 mg per mouse, o.p.) for indicated time periods. In total, 105 WT mice (male: female = 55: 50), 38 Tg-SwDI/B mice (male: female = 20: 18), and 5 5xFAD mice (male: female = 3: 2) were utilized in the study. No sexual impact on migrasome production has been observed (Fig. S3). All the mice were C57BL/6 J background and housed in a specific pathogen-free facility under 12 h light/dark cycle, temperature with 24 ± 2 °C, humidity between 30–70%, with access to food and water ad libitum. For all further experiments, pups and adult mice were euthanized using carbon dioxide.

### Cell depletion

Peripheral blood monocyte depletion was carried out according to published procedure^35^. Clodronate liposomes (Liposoma, 10 ml/kg, i.p.) was administered to 12-week-old Tg-SwDI/B mice every 3 days to deplete peripheral monocytes/macrophages prior to sacrifice at day 7. Depletion efficacy was confirmed with flow cytometric analysis.

For microglia depletion, PLX5622 was supplied to 12-week-old Tg-SwDI/B mice in the diet at 1200 PPM (1200 mg/kg of chow), starting 7 days prior to sacrifice^36^. Depletion efficacy of was confirmed with immunostaining.

Perivascular macrophage depletion was carried out according to published procedure^9^. 12-week-old Tg-SwDI/B mice were anesthetized with isoflurane and stereotaxically injected with 10 μL of clodronate-containing liposomes into the left lateral ventricle (coordinates from Bregma: anteroposterior, 0.2 mm; mediolateral, 1.2 mm; dorsoventral, 2.3 mm). Animals were sacrificed 7 days later and brains were processed for further analysis. Depletion efficacy of was confirmed with immunostaining.

### Transmission electron microscopy (TEM)

Fresh brain tissues were collected using a sharp blade within 1-3 min. The size of tissue block was <1 mm^3^. The 1 mm^3^ tissue blocks were transferred into an EP tube with fresh TEM fixative for further fixation, which was fixed at 4 °C for preservation and transportation. Micro-structure of tissue was observed after standard TEM sample preparation procedure with a TEM of HITACHI HT7800/HT7700. The membrane structures on separated migrasomes were observed by TEM after negative staining of the specimen with 3% phosphotungstic acid.

### Immune electron microscopy (IEM)

Fresh brain tissues were collected using a sharp blade within 1–3 min. The size of tissue block was <1 mm^3^. The 1 mm^3^ tissue blocks were transferred into an EP tube with fresh IEM fixative for further fixation, which was fixed at 4 °C for preservation and transportation. The IEM sample was prepared using rabbit anti-CD5L antibody (Immunoway YN1573, 1:1000). Samples were observed after standard IEM sample preparation procedure with a TEM of HITACHI HT7800 / HT7700. The 10 nm black golden particles were regarded as positive signals.

### Flow cytometric analysis

Flow cytometric analysis of cells was performed using a FACS flow cytometer (BD Biosciences, San Jose, CA). Flow cytometric analysis of migrasome from plasma was performed with a flow cytometer (Agilent, NovoCyte Advanteon). Peripheral blood of patients/HC or mice were isolated from mice with heparin based anticoagulation. Red blood cells in peripheral blood were removed with ACK lysis buffer. Leukocytes were washed with PBS and subjected to surface labeling. The following surface markers were used: APC/CY7 conjugated anti human CD14 (Biolegend 367108, clone: G10F5, 1:400), PerCP/CY5.5 conjugated anti human CD66b (Biolegend 305108, clone: 63D3, 1:400), FITC conjugated anti human CD3 (Biolegend 317306, clone: OKT3, 1:400), AF700 conjugated anti human CD19 (Biolegend 302226, clone: H1B19, 1:400), FITC conjugated anti human CD42b (Biolegend 303903, clone: HIP1, 1:400), PerCP conjugated anti mouse CD45 (Biolegend 103132, clone: 30-F11, 1:400), FITC conjugated anti mouse CD3 (Biolegend 100204, clone: 17A2, 1:400), APC/CY7 conjugated anti mouse CD3 (Biolegend 100220, clone: 17A2, 1:400), PE conjugated anti mouse CD19 (Biolegend 152408, clone: 1D3/CD19, 1:400), PE/CY7 conjugated anti mouse/human CD11b (Biolegend 101216, clone: M1/70, 1:400), BV421 conjugated anti mouse F4/80 (Biolegend 123132, clone: 8M8, 1:400), and APC/CY7 conjugated anti mouse Ly6G (Biolegend 108424, clone: RB8-8C5, 1:400). After being washed with PBS, cells were fixed and permeabilized (Invitrogen, Intracellular Fixation & Permeabilization Buffer Set), then stained with intracellular antibodies. To estimate migrasome productivity of leukocytes, migrasome markers were labeled with primary antibodies for overnight. The following primary antibodies were used: rabbit anti-TSPAN4 (Abcam ab181995, 1:200), rabbit anti-TSPAN7 (Proteintech 18695-1-AP, 1:200), rabbit anti-Tspan9 (Proteintech 21983-1-AP, 1:200), rabbit anti-NDST1 (Proteintech 26203-1-AP, 1:200). Aβ40 phagocytosis by macrophages was labeled with the primary antibodies of mouse anti-Amyloid beta (aa 1-40) (R&D MAB96181R-SP, 1:200). Secondary antibodies of anti-rabbit Alexa Fluor 647 (Abcam, 1:1000) and anti-mouse Alexa Fluor 488 (Proteintech, 1:1000) were then used to conjugate the primary antibodies. Fluorochrome compensation was performed with single stained OneComp eBeads (Thermo Fisher eBioscience). Data analysis was performed using FlowJo software (FlowJo, version 10.0, Ashland, OR).

### Cell lines

Mouse macrophage cell line Raw 264.7 cell line HEK293T was purchased from ATCC and mouse brain vessel pericyte (MBVP) cell line was purchased from NEWGAINBIO. To construct CD5L deficiency macrophage, Raw 264.7 was transduced with gRNA-expressing lentivirus, and knock-out cells were collected by cell sorting.

### Human monocyte enrichment and macrophage differentiation

Mono-nucleus cells were isolated from peripheral blood of healthy adults (age = 18–40 y) with Ficoll (GE healthcare) -mediated density gradient centrifugation (18 °C, × 800 *g*, 15 min with low acceleration and deceleration (speed = 3)). Mononucleus cells were allowed to subside in culture plates for 15 min. Lymphocytes were then removed with 3 washes of the culture with PBS. Monocytes were thus enriched and subjected to further experiments. For macrophage differentiation, monocytes adhered 4 h on tissue-culture plates were cultured in RPMI medium 1640 containing 10% FBS, 1% L-glutamine, and 1% penicillin/streptomycin (Life Technologies) 10% male human serum and 30 ng/ml macrophage colony stimulating factor (M-CSF, Peprotech). Medium was replaced every 3 days until day 7, when the cells were prepared for further experiments^37^.

### Primary mouse bone marrow derived macrophage culture

Primary bone marrow derived macrophage culture was prepared by isolated cells from femurs and tibia of healthy WT C57/BL6 wild type donors (8–12 weeks old). Macrophage precursors were cultivated and differentiated to macrophages for 6 days in MCSF-conditioned RPMI1640 medium containing 10% FBS and 1% penicillin-streptomycin (PS).

### Primary mouse microglia and astrocyte culture

Primary microglia- and astrocyte-enriched culture were prepared from the whole brains of 1- to 3-day-old WT mouse pups^38^. Briefly, brain tissue of mouse pups was isolated and digested then seeded in 75 mm^2^ flasks with DMEM/F12-based glia cell medium. Microglia were isolated from the mix-glia culture with 180 rpm shaking for 1 h. Oligodendrocyte lineage cells were obtained by shaking the culture with 200 rpm overnight. The adhesive cells were removed from the flasks with 0.25% Trypsin and were allowed with 15 min attachment. Astrocytes were thus enriched after removing the non-attached cells.

### Primary mouse brain blood vessel endothelial cell culture

Primary mouse brain microvessel endothelial cells were isolated according to the reported methodology^39^. Briefly, 8-week-old healthy C57/BL6 WT mice were sacrificed and brain tissue was isolated. The tissue sample was fragmented into small pieces and digested with Liberase Blendzyme (0.625 mg/ml, Roche) at 37 °C on a rotator for 1 h. The brain cells were then seeded in Collagen type I coated flasks in EndoPM culture medium (1% Endothelial Cell Growth Supplement (ECGS), 1% antibiotic solution, and 5% fetal bovine serum in Endothelial Cell Medium). The cellular suspension was maintained in the incubator for 14 days. Endothelial cells were then enriched with magnetic sorting with anti-CD31 microbeads (Miltenyi) with auto-MACS (Miltenyi) and expanded before subjected to further experiments.

### Construction of in vitro endothelial barrier

The in vitro endothelial barrier was constructed by seeding brain blood vessel endothelial cells on a cell culture insert (LABSELECT 14312, pore diameter = 0.4 μm) overnight. At 2 h after migrasome administration, trans-endothelial electrical resistance (TEER) of the barrier was measured with Electrical Resistance System (Millipore ERS-2), and culture medium was collected for LDH release measurement. Finally, the intrinsic TEER value of the monolayer is calculated as the electrical resistance of the barrier multiplied by the filter area^40^. Medium in the upper chamber was then renewed in addition with Fluorescein sodium (NaF, Sigma-Aldrich F6377, 250 μM, Ex/Em = 460/515 nm) and permeability to NaF of the barrier was calculated as the fluorescent intensity of NaF in the lower chamber as measured with a 96-well plate reader (Biotek Synergy H1MF). In indicated experiments, human complement (Quidel A113, 10% in culture medium) was administered to the endothelial barrier or endothelial cells as 10% in culture system. In indicated experiments, CD5L was treated to the endothelial barrier (100 ng/ml) with or without anti-CD36 monoclonal antibodies (Abcam ab23680, 0.1 μg/ml).

### Isolation of migrasomes from culture cells and plasma samples

Cells were grown in 150 mm dishes coated with 0.1 μg/ml fibronectin (BD 354008) in complete macrophage culture medium for 12 h. The cells and migrasomes on plates were digested with 0.25% trypsin and collected in 50 ml tubes. All subsequent manipulations were performed at 4 °C. Cells and large debris were removed by centrifugation at × 1000 *g* for 10 min followed by × 4000 *g* for 20 min. Blood samples were collected in anticoagulation tubes using standard venepuncture protocols. Large debris was removed by centrifugation at × 1000 *g* for 10 min followed by × 4000 *g* for 20 min. Crude migrasomes were then collected as the pellet by centrifugation at × 20000 *g* for 30 min. The crude migrasome pellets were washed with PBS and centrifuged down again at × 20000 *g* for 30 min for two times before further analysis or in vitro treatment. The pellets were subjected to quantify protein content. The sample preparation methodology was compatible in western blot analysis and mass spectrometry. The particle diameter and zeta potential of separated migrasomes were measured by a Particle Analyzer Litesizer 500 (Anton Paar, Austria).

### Migrasome productivity analysis

In macrophage cultures, migrasomes were outlined with wheat germ agglutinin (WGA, Invitrogen W7024, 1 μg/ml, 15 min) which is a widely used lectin that binds to sialic acid and N-acetylglucosaminyl residues in cell membrane. In CAA patients and murine models, since migrasomes were adhesive to blood vessel wall and could hardly be isolated in peripheral blood, migrasome productivity in cells was estimated with the expression of migrasome membrane proteins including Tetraspanin (TSPAN) 4, TSPAN7, TSPAN9, N-Deacetylase And N-Sulfotransferase 1 (NDST1), Rho Associated Coiled-Coil Containing Protein Kinase 1 (ROCK1), Chromosome 3 Open Reading Frame 64 (C3ORF64), Plasma glutamate carboxypeptidase (PGCP) and Phosphatidylinositol Glycan Anchor Biosynthesis Class K (PIGK) by immunostaining, flow cytometry and western blot.

### BBB integrity assessment

BBB integrity in mice was estimated with BBB permeability analysis. In BBB permeability assessment, a single dose of 3kDa-Dextran (3kDa-Dex, either labeled with Alexa fluor −488 or −555, Invitrogen D3306, Invitrogen D3308) was intravenously injected to mice (10 mg/kg) at 90 min before sacrifice. After collection of peripheral blood, eyeballs of mice were dissected and perfusion with PBS (10 ml) then 4% paraformaldehyde (Biosharp BL539A, 10 ml) was performed. Two eyeballs of each recipient were ground in 500ul PBS. Fluorescent intensity of the 3kDa-Dextran in plasma and the eyeball extract was measured with a 96-well plate reader (Biotek Synergy H1MF). Extravasation index of 3kDa-Dextran in brain parenchyma of the recipients was calculated as the ratio of extra- to intra-vessel 3kDa-Dextran MFI (Image J, NIH) which was further normalized to that of the MOCK group.

### Migrasome structure destruction (ultrasound)

Intrinsic construction of migrasomes was constructed by ultrasound (600w, 10 min, 3 s on/off). Protein and RNA concentration of migrasomes was preserved in the ultrasonic procedure.

### Removal of Aβ40 and Aβ42 from plasma

Anti-Aβ40 monoclonal antibodies (Invitrogen, 44348 A, 1 μg in 1 ml plasma) and anti-Aβ42 monoclonal antibodies (Abcam, ab180956, 1 μg in 1 ml plasma) were applied in the plasma of CAA patients and healthy donors. After incubation for overnight at 4 °C on a rotator, Pierce Protein A/G Magnetic Beads (Thermo Fisher 88802, 3ul in 1 ml plasma) were added into the neutralization system and incubated for another 2 h at 4 °C. Aβ40 and Aβ42 was then precipitated from plasma by removing the beads with a magnetic stand. Efficiency of Aβ40 and Aβ42 removal was validated with ELISA (Fig. S3B).

### Amyloid Oligomer Preparation

Basic stock solution of monomeric Aβ40 (MCE, HY-P0265A) and Aβ42 (MCE, HY-P1363) were prepared by dissolving one tube of purified Aβ40 and Aβ42 in 25 mM NaOH solution, followed by filtered through a 0.22 μm centrifuger to remove large aggregates. Stock Aβ basic solution was diluted by adding phosphate buffer solution (PB). The final concentration of PB was 25 mM containing 0.02% NaN3 (w/v), and the pH was set at 7.4. To prepare Aβ oligomers, the solution was kept at 4°C for 7d^41^. Aβ16 (MCE, HY-P1466) and Aβ22-35 (MCE, HY-P1474A) were obtained commercially.

### Lentiviral infection of macrophages

To knock down *Tspan4* expression in BMDM, shRNA of *Tspan4* was inserted into the lentiviral transfer vector PTSB-SH-copGFP-2A-PURO; TSB181089-2 C-terminal. Four shRNA was designed and two efficiently knocked down *Tspan4* expression, whose sequences were: *Tspan4* ^KD1^: 5′-ACCGGTGCCGTGCTACGAGACGGTGAACTCGAGTTCACCGTCTCGTAGCACGGCTTTTTGAATTC-3′; *Tspan4*^KD2^: 5′-ACCGGTGTACCTCATGTTCGCCTTCAACTCGAGTTGAAGGCGAACATGAGGTACTTTTTGAATTC-3′. Overexpression of *Tspan4* was fulfilled by inserting *Tspan4* cDNA into the lentiviral transfer vector of TK-PCDH-copGFP-T2A-Puro. The inserted sequence of *Tspan4* cDNA was: ATGGCGCGCGGCTGCCTCCAGGGCGTCAAGTACCTCATGTTCGCCTTCAACCTGCTCTTCTGGCTGGGTGGCTGTGGTGTCCTGGGTGTTGGCATCTGGTTGGCTGCCACACAGGGAAACTTTGCCACCTTATCATCCTCATTTCCATCCTTGTCGGCTGCCAACCTGCTCATCGTCACCGGGACCTTCGTCATGGCCATCGGCTTCGTGGGCTGCATTGGGGCCCTCAAGGAGAACAAGTGCCTACTGCTCACTTTCTTTGTGCTGCTGCTGCTAGTGTTCCTGCTGGAAGCCACCATTGCTGTGCTCTTCTTTGCCTACAGTGACAAGATTGACAGTTATGCCCAACAAGACCTGAAGAAGGGCCTGCATCTGTATGGCACACAGGGCAACGTGGGCCTCACCAATGCCTGGAGCATCATCCAGACTGATTTCCGATGCTGTGGAGTTTCCAATTACACTGATTGGTTTGAGGTATACAATGCCACTCGTGTGCCTGACTCCTGCTGTCTGGAGTTCAGTGATAGCTGTGGGTTACATGAACCTGGTACCTGGTGGAAGTCGCCCTGTTATGAGACAGTGAAGGCCTGGCTCCAGGAGAACCTGCTAGCTGTGGGCATCTTTGGACTGTGCACGGCACTGGTGCAGATTCTGGGCCTCACCTTCGCTATGACCATGTACTGCCAGGTGGTAAAGGCGGACACCTACTGTGCATAG.

The constructed transfer vectors were transformed into DH5α E. coli and then isolated using the Endo-free Plasmid Maxi Kit (Omega). A plasmid mixture containing psPAX2, pMD2.G and the transfer vector was suspended in OPTI-MEM and PEI MAX 40 K (Polysciences 24765-1) was applied as transfection reagent. The plasmids containing OPTI-MEM was then added to 293 T cells and allowed incubation for 8 h before switching to fresh culture medium. Supernatant was collected at 48 h after transfection and centrifuged at × 800 *g* for 10 min to further eliminate cell debris. The lentivirus containing medium was treated to BMDM for 8 h before switching to fresh culture medium until 48 h to fulfill shRNA dependent *Tspan4* knockdown or *Tspan4* overexpression, whose efficiency was evaluated with RT-PCR or western blot.

### Cell viability analysis

Endothelial viability was assessed with the Lactate Dehydrogenase (LDH) assay (Invitrogen, C20300) and/or the LIVE/DEAD Viability analysis (ThermoFisher, L7013) based on SYTO10 (green, outlined both lived and dead cells) and Ethidium homodimer-2 (red, indicating dead or injured cells) staining according to instructions from manufacturers. Activation of Caspase signaling was evaluated with western blot. To stain for cell death markers, APC-conjugated Annexin V (Biolegend, 640919) was incubated at 4 °C for 15 min with the endothelial cells. PI was added right before acquisition for flow cytometry. The percentage of apoptotic or necrotic cells, as defined by Annexin V^+^ and/or Annexin V^-^PI^+^ cells, were obtained using FlowJo software (FlowJo, version 10.0, Ashland, OR).

### Western blot

Protein was extracted with RIPA lysis buffer (Sigma) from cells or migrasomes. A total amount of 40ug protein of each sample was applied to western blot experiments. Western blot was performed with standard SDS-polyacryamide gel electrophoresis method and ECL western HRP substrate (Affinity Biosciences KF8001-500). The following primary antibodies were used: rabbit anti-TSPAN4 (Abcam ab181995, 1:1000), rabbit anti-TSPAN7 (Proteintech 18695-1-AP, 1:1000), rabbit anti-TSPAN9 (Proteintech 21983-1-AP, 1:1000), rabbit anti-NDST1 (Proteintech 26203-1-AP, 1:1000), rabbit anti-ROCK1 (Invitrogen PA5-22262, 1:1000), rabbit anti-C3ORF64 (Proteintech 27595-1-AP, 1:1000), rabbit anti-PIGK (Proteintech 15151-1-AP, 1:1000), rabbit anti-PGCP (Proteintech 16601-1-AP, 1:1000), mouse anti-caspase1 (Santa cruz biotechnology sc-56036, 1:1000), rabbit anti-Caspase 3 (Proteintech 19677-1-AP, 1:1000), rabbit anti-Caspase 7 (Proteintech 27155-1-AP, 1:1000), rabbit anti-Caspase 8 (Proteintech 13423-1-AP,1:1000), rabbit anti-Caspase 9 (Proteintech 10380-1-AP, 1:1000), rat anti-Caspase 11 (Cell Signaling Technology 14340 S, 1:1000), rabbit anti-Caspase 12 (Cell Signaling Technology 35965 S, 1:1000), rabbit anti-ZO1 (Invitrogen 617300, 1:1000), rabbit anti-CD5L (Immunoway YN1573,1:1000), rabbit anti-HBB (Proteintech 16216-1-AP, 1:1000), rabbit anti-HBA (Proteintech 14537-1-AP, 1:1000), rabbit anti-KRT1 (Proteintech 16848-1-AP, 1:1000), mouser anti-CRP (Proteintech 66250-1, 1:1000), rabbit anti-CD55 (Abclonal A1689, 1:1000), rabbit anti-CD59 (Proteintech 26580-1-AP,1:1000), mouse anti-GAPDH (Proteintech 60004-1-Ig, 1:1000). Immunoreactivity was assessed with Image J (NIH).

### Immunofluorescence staining

In in vivo experiments, mice were sacrificed at indicated time points. After sufficient perfusion with 10 ml of PBS and 10 ml of 4% paraformaldehyde, brains were removed and cut into coronal sections (25 μm) on a frozen microtome (CM1950). In in vitro experiments, macrophages were seeded on coverslips coated with poly-L-lysine (Biosharp BS005). After treatment, cells were fixed with 4% paraformaldehyde. Brain sections or fixed BMDM were washed and incubated with primary antibodies overnight in PBS containing 0.03% Triton-X100 and 3% BSA. After wash, sections or cells were incubated with secondary antibodies for 1 h at room temperature. The following primary antibodies were used: rat anti-CD31 (Bioscience 550274, 1:50), goat anti-iba1 (Abcam ab178847, 1:500), rabbit anti-TSPAN4 (Abcam ab181995, 1:500), mouse anti-Aβ40 monoclonal antibodies (R&D MAB96181R-SP, 1:500), rabbit anti-ZO1 (Invitrogen 617300, 1:500), goat anti-CD5L (R&D AF2834, 1:500), rabbit anti-C5b-9 (Bioss bs-2673R, 1:1000), rabbit anti-MBP (Proteintech 10458-1-AP, 1:500), Mouse anti-NFH (Proteintech 60331-1-Ig, 1:500). The following secondary antibodies were applied: anti-rat secondary antibody conjugated with Cy3 (Jackson ImmunoResearch Laboratories 112-545-003, 1:1000), anti-goat secondary antibody conjugated with Cy3 (Jackson ImmunoResearch Laboratories 305-165-003, 1:1000), anti-rabbit secondary antibody conjugated with Alexa Fluor 405 (Jackson ImmunoResearch Laboratories 111-475-003, 1:1000), anti-rabbit secondary antibody conjugated with Cy3 (Jackson ImmunoResearch Laboratories 111-165-003, 1:1000), anti-rabbit secondary antibody conjugated with 488 (Jackson ImmunoResearch Laboratories 111-545-003,1:1000), anti-mouse secondary antibody conjugated with Alexa Fluor 488 (Invitrogen A-11059, 1:1000), and anti-mouse secondary antibody conjugated with Alexa Fluor 405 (Abcam ab175658,1:1000). The coverslips or slices were finally mounted with anti-fade fluorescence mounting medium (Abcam ab104135), or mounting medium containing DAPI (Abcam ab104139) to locate nucleus when indicated. Phalloidin (Invitrogen A12379, 1:3000) was used to label Actin to outline cells when indicated. Images were captured with a confocal microscopy (Leica TSC SP8) and processed with NIH Image J software by a blinded observer for the unbiased counting of automatically recognized cells and mean fluorescent intensity calculation.

### Enzyme linked immunosorbent assay (ELISA)

Plasma of patients and HC, mouse migrasome recipients, and CAA murine models was collected via centrifugation of peripheral blood (× 15000 *g*, 15 min, 4 °C) and stored at −80 °C before examination. Concentration of mouse NEFL (FineTest, EM1688), mouse C5b-9 (FineTest, EM1392), mouse CD5L (Boster, EK1414), human CD5L (MEIMIAN, MM-50587H1), human Aβ40 (CUSABIO, CSB-E08299h), human Aβ42 (MAI Bio, LM-2685H), and human C5b-9 (FineTest, EH3858) in plasma was assessed with commercial kits according to instructions of manufacturers.

### Bulk RNA sequencing

Two replicates for each group of endothelial cells were seeded at 5 × 10^6^ cells in 10 cm dishes and treated as described above. The cells were collected and total RNA was isolated using the RNeasy kit (Qiagen) according to the manufacturer’s instructions. RNA integrity was assessed using the RNA Nano 6000 Assay Kit of the Bioanalyzer 2100 system (Agilent Technologies, CA, USA). A total amount of 1 μg RNA per sample was used as input material for the RNA sample preparations. Briefly, mRNA was purified from total RNA using poly-T oligo-attached magnetic beads. Fragmentation was carried out using divalent cations under elevated temperature in first strand synthesis reaction buffer (5×). First strand cDNA was synthesized using random hexamer primer and M-MuLV Reverse Transcriptase. Second strand cDNA synthesis was subsequently performed using DNA polymerase I and RNase H. Remaining overhangs were converted into blunt ends via exonuclease/polymerase activities. After adenylation of 3′ ends of DNA fragments, adaptor with hairpin loop structure was ligated to prepare for hybridization. To select cDNA fragments of preferentially 370~420 bp in length, the library fragments were purified with AMPure XP system (Beckman Coulter, Beverly, USA). Then PCR was performed with Phusion High-Fidelity DNA polymerase, universal PCR primers and Index (X) Primer. At last, PCR products were purified (AMPure XP system) and library quality was assessed on the Agilent Bioanalyzer 2100 system. The clustering of the index-coded samples was performed on a cBot Cluster Generation System using TruSeq PE Cluster Kit v3-cBot-HS (Illumia) according to the manufacturer’s instructions. After cluster generation, the library preparations were sequenced on an Illumina Novaseq platform and 150 bp paired-end reads were generated. Raw data (raw reads) of fastq format were firstly processed through in-house Perl scripts. In this step, clean data (clean reads) were obtained by removing reads containing adapter, reads containing ploy-N and low-quality reads from raw data. At the same time, Q20, Q30 and GC content the clean data were calculated. Reference genome and gene model annotation files were downloaded from genome website directly. Index of the reference genome was built and paired-end clean reads were aligned to the reference genome using Hisat2 v2.0.5. FeatureCounts v1.5.0-p3 was used to count the reads numbers mapped to each gene. And then FPKM of each gene was calculated based on the length of the gene and reads count mapped to this gene. Differential expression analysis of two groups was performed with the software of ***R*** using the “DESeq2” package (1.20.0). The *P*-values were adjusted using the Benjamini and Hochberg’s approach for controlling the false discovery rate. Genes with an adjusted *P* < 0.05 found by “DESeq2” were assigned as differentially expressed genes. Subsequently, Gene Ontology (GO) analysis was conducted to down-regulated genes with the software of ***R*** using the “clusterProfiler” package. Transcriptional level of the tight junction related proteins was visualized with heat map and clustered with the software of ***R*** using the “pheatmap” package. RNAseq data has been deposited to GEO under accession GSE222419.

### Isobaric tags for relative and absolute quantification (ITRAQ)

Frozen samples were lysed in RIPA lysis buffer (0.1%SDS, 1%Triton X-100, 150 mM NaCl, 1 mM EDTA, 0.5 mM EGTA, 50 mM Tris·HCl, pH7.4 and protease inhibitor cocktail) and one tablet of phosphatase inhibitor cocktail (PhosSTOP, Roche), subsequently homogenized by sonication (Scientz, 2 s on; 3 s off) on ice. The homogenate was cleared using centrifugation at 12000 rpm for 15 min at 4 °C. Supernatants were transferred into clean tubes prior to the determination of protein concentrations by BCA assays. Aliquots of lysates were mixed with 200ul of 8 M urea in Nanosep Centrifugal Devices (PALL). The device was centrifuged at × 14000 *g* at 20 °C for 20 min. All following centrifugation steps were performed applying the same conditions allowing maximal concentration. The concentrate was diluted with 200ul of 8 M urea in 0.1 M Tris-HCl, pH8.5, and the device was centrifuged. Proteins were reduced with 10 mM DTT for 2 h at 56 °C. Subsequently, the samples were incubated in 5 mM iodoacetamide for 30 min in the dark to block reduced cysteine residues followed by centrifugation. The resulting concentrate was diluted with 200ul 8 M urea in 0.1 M Tris-HCl, pH = 8.0, and concentrated again. This step was repeated 2 times, and the concentrate was subjected to proteolytic digestion overnight at 37 °C. The digests were collected by centrifugation. The lyophilized peptide fractions were re-suspended in ddH_2_O containing 0.1% formic acid, and 2ul aliquots of which was loaded into a nanoViper C18 (3μm, 100 A) trap column. The online chromatography was performed on the Easy-nLC 1200 system (ThermoFisher). The trapping and desalting procedure were carried out with a volume of 20 μL 100% solvent A (0.1% formic acid). Then, an elution gradient of 8-38% solvent B (80% acetonitrile, 0.1% formic acid) in 60 min was used on an analytical column (50 μm × 15 cm C18-3μm 100 A). DDA (data-dependent acquisition) mass spectrum techniques were used to acquire tandem MS data on a ThermoFisher Q Exactive mass spectrometer (ThermoFisher, USA) fitted with a Nano Flex ion source. Data was acquired at an ion spray voltage of 1.9 kV, and an interface heater temperature of 275 °C. The MS was operated with FULL-MS scans. For DDA, survey scans were acquired in 250 ms and up to 20 production scans (50 ms) were collected. Only spectra with a charge state of 2–4 were selected for fragmentation by higher-energy collision energy. Dynamic exclusion was set for 25. The MS/MS data were analyzed for protein identification and quantification using Proteome discoverer (v2.5.0400). The local false discovery rate was 1.0% after searching against Mus musculus protein database with a maximum of two missed cleavages and one missed termini cleavage. Precursor and fragment mass tolerance were set to 10ppm and 0.05 Da, respectively. Differential expression analysis of two groups was performed with the software of ***R*** using the “limma” package (3.44.3). The statistically significant difference was set as *P* < 0.05 and FC > 1.5 or FC < 0.67 by “limma”, and these genes were assigned as the differential expression genes which were visualized with the volcano plot by the “ggplot2” package. GO analysis was conducted to up-regulated proteins of two group, or the top 100 proteins according to the expression levels of each group with the software of ***R*** using the “clusterProfiler” package. GO-BP correlation of PBS-M and Aβ40-M was performed with the software of ***R*** using the “Venn diagram” package. The mass spectrometry proteomics data have been deposited to the ProteomeXchange Consortium via the PRIDE partner repository with the dataset identifier PXD039329.

### Statistic analysis

Test of normality was performed before the parametric analysis of Student’s *t* test and one-way ANOVA followed by Tukey’s post-test. GraphPad Prism software (version 8.0) was used for statistical analysis. Results were considered significant at *P* < 0.05. *Student’s t test.* Unpaired parametric *t* test (two-tailed) was performed in data comparison of two groups. Error bar represents standard error of mean (SEM). *One-way ANOVA*. No matching or pairing ANOVA was performed in data comparison of three groups or more. Result was corrected for multiple comparisons using statistical hypothesis testing (Dunnett). Error bar represents SEM. *Spearman Correlation*. Correlation between every pair data sets was computed with Spearman correlation coefficients. The value of *r* was visualized with heatmap. *ROC analysis*. In the current study, ROC analysis has been used to evaluate the diagnostic efficacy of monocyte TSPAN4 expression, plasma Aβ40 level, plasma Aβ40/Aβ42 ratio and plasma NeFL concentration to distinguish CAA patients (TRUE) and HC (FALSE), or to estimate the identification efficiency of plasma C5b-9 level to distinguish patients with CAA (TRUE) from healthy donors (FALSE) and from those with aCSVD or CADASIL (FALSE). Binary logistic regression was performed with the dependent parameter of grouping with the indicated covariates in SPSS. Probabilities were calculated in SPSS and subjected to GraphPad Prism for ROC curve description.

### Reporting summary

Further information on research design is available in the Nature Portfolio Reporting Summary linked to this article.

## Supplementary information

Supplementary Information

Reporting Summary

## Source data

Source Data

## Data Availability

All data needed to evaluate the conclusions in the paper are present in the paper and/or the Supplementary Materials. Source data are provided with this paper. All the databases/datasets used in the study along with appropriately accessible links/accession-codes. RNAseq data has been deposited to GEO under accession GSE222419. The mass spectrometry proteomics data have been deposited to the ProteomeXchange Consortium via the PRIDE partner repository with the dataset identifier PXD039329. Source data are provided with this paper.
