# Author Correction: Macrophage lineage cells-derived migrasomes activate complement-dependent blood-brain barrier damage in cerebral amyloid angiopathy mouse model

**DOI:** 10.1038/s41467-025-67562-2

**Published:** 2025-12-15

**Authors:** Mengyan Hu, Tiemei Li, Xiaomeng Ma, Sanxin Liu, Chunyi Li, Zhenchao Huang, Yinyao Lin, Ruizhen Wu, Shisi Wang, Danli Lu, Tingting Lu, Xuejiao Men, Shishi Shen, Huipeng Huang, Yuxin Liu, Kangyu Song, Banghao Jian, Yuxuan Jiang, Wei Qiu, Quentin Liu, Zhengqi Lu, Wei Cai

**Affiliations:** 1 Department of Neurology, Mental and Neurological Disease Research Center, The Third Affiliated Hospital of Sun Yat-sen University, Guangzhou, China; 2 Guangdong Provincial Key Laboratory of Brain Function and Disease, Guangzhou, China; 3 Center of Clinical Immunology, Mental and Neurological Disease Research Center, The Third Affiliated Hospital of Sun Yat-sen University, Guangzhou, China; 4 Department of Neurosurgery, The Third Affiliated Hospital of Sun Yat-Sen University, Guangzhou, China; 5 Sun Yat-sen University Cancer Center, State Key Laboratory of Oncology in South China, Guangzhou, China

**Keywords:** Neurovascular disorders, Monocytes and macrophages, Complement cascade

Correction to: *Nature Communications* 10.1038/s41467-023-39693-x,published online 04 July 2023

The original version of this Article contained incorrect representative microscopy images in Fig. 7e (MBP staining) and Fig. S15g (ZO-1 staining). The incorrect representative images were the image for the STR, PBS-M group in Fig. 7e, and the image for the CD5L-KO, Aβ40-M group in Fig. S15g. These incorrect images were not used for any analyses. The figures are now amended in the HTML and PDF versions of the article.

