# Supplementary material for: Macrophage lineage cells-derived migrasomes activate complement-dependent blood-brain barrier damage in cerebral amyloid angiopathy mouse model
Source: Nat Commun. 2023 Jul 4;14:3945. doi: 10.1038/s41467-023-39693-x (PMC10319857; doi:10.1038/s41467-023-39693-x)
Supplement: Supplementary file 1 — Supplementary Information [file 41467_2023_39693_MOESM1_ESM.pdf]

Supplementary Material

Supplementary Figures

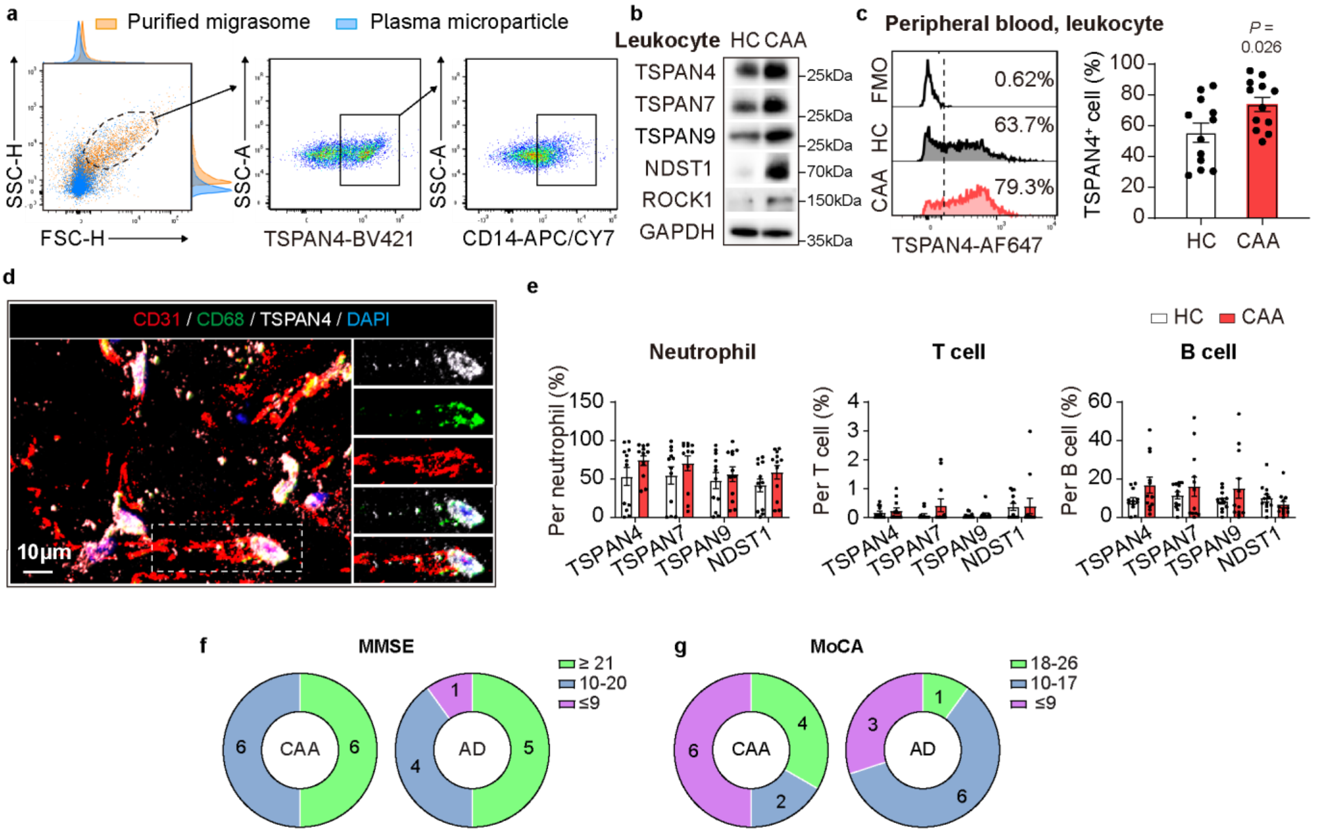

Figure S1. Excessive migrasome production in CAA is not observed in immune cells other than macrophages. (**a**) Gating strategy of plasma migrasomes in flow cytometric analysis. Purified migrasomes derived from BMDM *in vitro* were set as FSC/SSC scale standard. Migrasomes were further identified as TSPAN4^{+} particles. (**b-c**) Leukocytes in peripheral blood of CAA patients and age- and sex-matched healthy controls (HC) were isolated after red blood cell lysis. (**b**) Representative western blot images showing the elevated migrasome markers expression in leukocytes of CAA patients. Data shown are representative of 3 biologically independent experiments. (**c**) Migrasome marker expression in circulating leukocytes of the recruited cohort was analyzed with flow cytometry. TSPAN4 expression in CAA patients and HC. N = 12 in CAA groups. N = 12 in HC group. Data are presented as mean values ± SEM with the indicated significance (by two-tailed Student's t test). (**d**) Skin autopsy samples of CAA patients were subjected to immunostaining of CD68 (green), TSPAN4 (grey) and CD31 (red). Data shown are representative of 3 biologically independent experiments. (**e**) Migrasome marker expression in neutrophil (CD66b^{+}), T cell (CD3^{+}) and B cell (CD19^{+}). N = 12 in CAA groups. N = 12 in HC group. Data are presented as mean values ± SEM, and no

statistically significant differences were observed (by two-tailed Student's *t* test). (**f-g**) MMSE (**f**) and MoCA (**g**) scoring of recruited CAA and AD patients. *N* = 12 in CAA group and *N*= 10 in AD group. Source data are provided as a Source Data file.

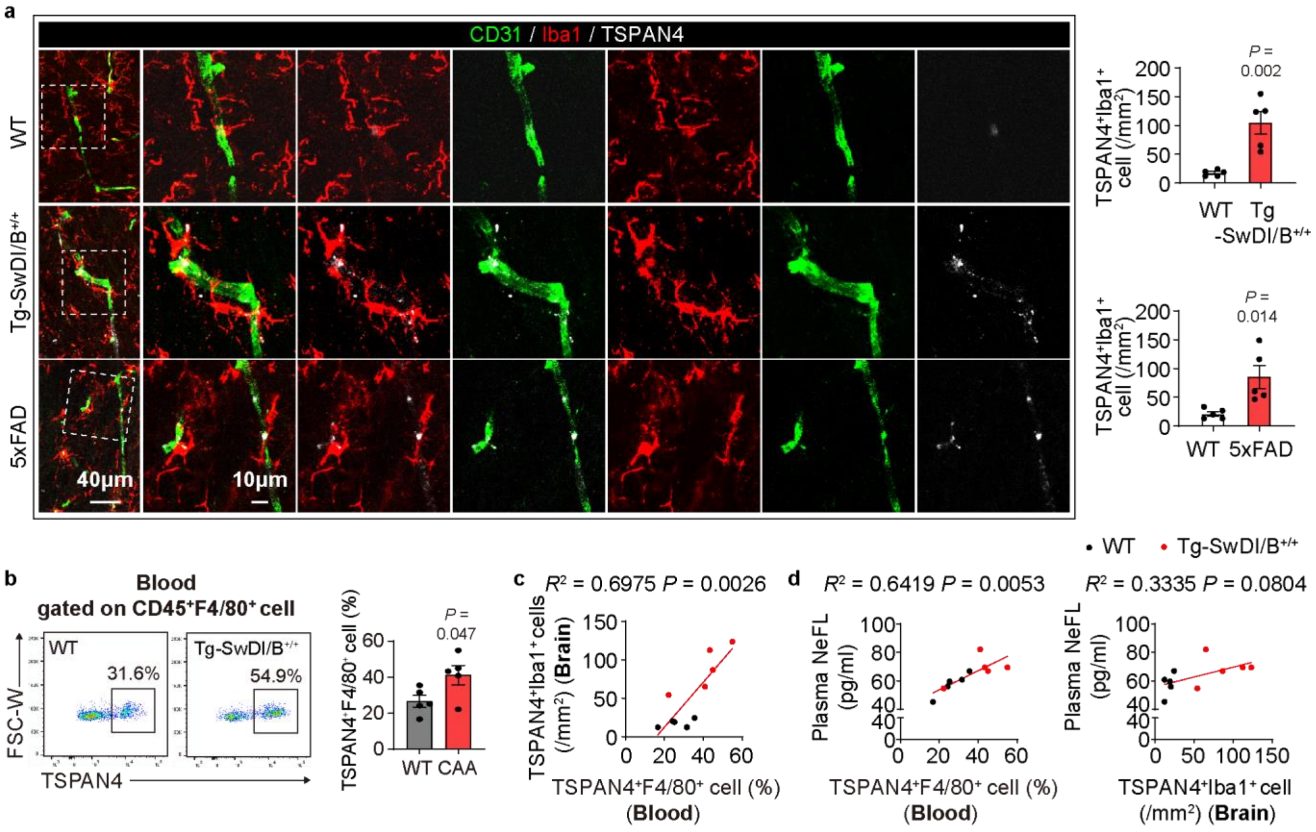

Figure S2. Migrasome production in CAA brains. Age- and sex-matched wild type (WT), Tg-SwDI/B$^{+/+}$ and 5xFAD mice were sacrificed at 24w of age. (**a**) Coronal brain sections of WT, Tg-SwDI/B$^{+/+}$ and 5xFAD mice were subjected to immunostaining of the microglia/macrophage marker Iba1 (red), the blood vessel marker CD31 (green) and the migrasome marker TSPAN4 (grey) and analyzed with confocal microscopy. The number of TSPAN4 expressing Iba1$^+$ cells in WT and CAA mice was calculated. N = 5 in both groups. Data are presented as mean values ± SEM with the indicated significance (by two-tailed Student's t test). (**b**) TSPAN4 expression in circulating monocytes/macrophages (CD45$^+$F4/80$^+$ cells) of Tg-SwDI/B$^{+/+}$ mice was assessed with flow cytometry. N = 5 in both groups. Data are presented as mean values ± SEM with the indicated significance (by two-tailed Student's t test). (**c**) Association of migrasome productivity (TSPAN4 expression) of brain microglia/macrophages (Iba1$^+$ cells) and blood monocytes/macrophages (CD45$^+$F4/80$^+$ cells) in WT (Black dots) and Tg-SwDI/B$^{+/+}$ mice (red dots) was evaluated with Spearman correlation analysis. The data is represented using numerical values along with a regression line, and R^2 and P value were presented. N = 5 in both groups. (**d**) Spearman correlation analysis for the association between plasma NeFL concentration and TSPAN4 expression of brain microglia/macrophages (Iba1$^+$ cells) (**Left**) or blood monocytes/macrophages (CD45$^+$F4/80$^+$ cells) in WT (Black dots) and Tg-SwDI/B$^{+/+}$ mice (red dots) (**Right**).

N = 5 in both groups. The data is represented using numerical values along with a regression line, and R^2 and *P* value were presented. Source data are provided as a Source Data file.

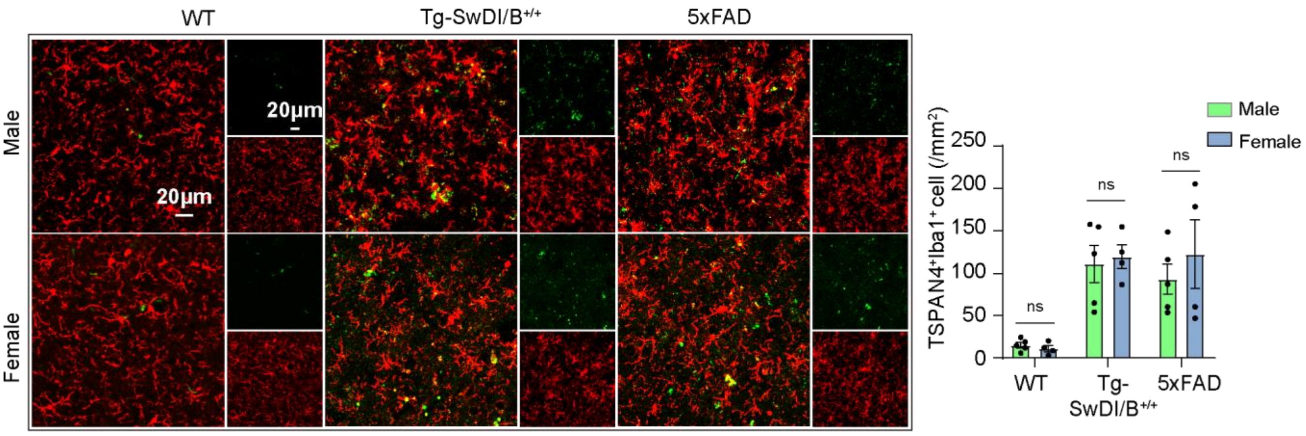

Figure S3. Sexual impact on migrasome production in CAA models. Age- and sex-matched wild type (WT), Tg-SwDI/B$^{+/+}$ and 5xFAD mice were sacrificed at 24w of age. Migrasome production in the brain microglia/macrophage of male (N = 5 in WT mice, N = 5 in Tg-SwDI/B$^{+/+}$ mice, N = 5 in 5xFAD mice) and female (N = 4 in WT mice, N = 4 in Tg-SwDI/B$^{+/+}$ mice, N = 4 in 5xFAD mice) CAA models was calculated. Data are presented as mean values ± SEM, and no statistically significant differences were observed (by two-tailed Student's *t* test). Source data are provided as a Source Data file.

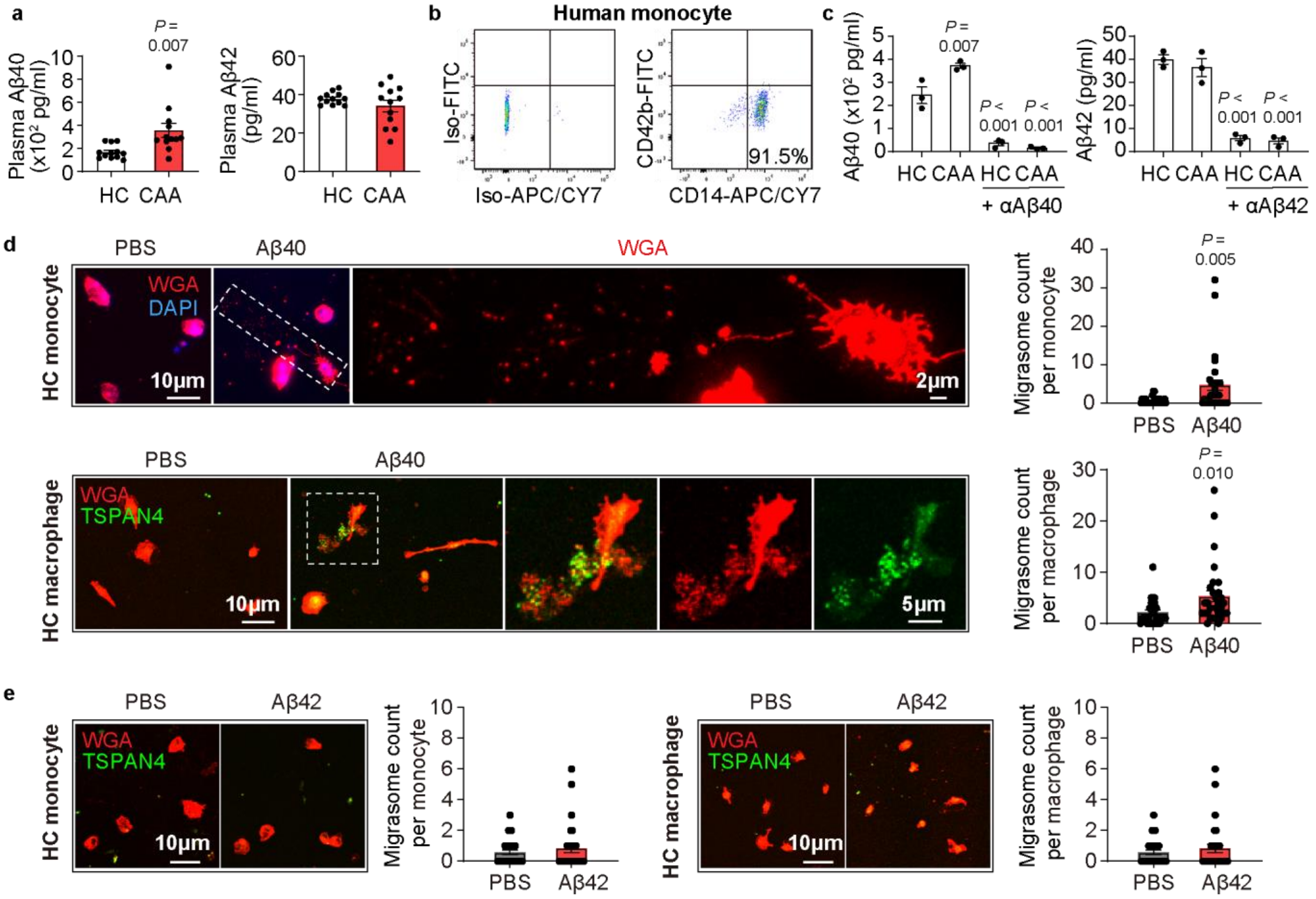

Figure S4. Aβ40 is a sufficient inducer of macrophage-derived migrasomes. (**a**) Concentration of Aβ40 and Aβ42 in CAA plasma. *N* = 12 in CAA groups. *N* = 12 in HC group. Data are presented as mean values ± SEM with the indicated significance (by two-tailed Student's *t* test). (**b**) Human monocytes were isolated from peripheral blood of healthy donors. Purity of the isolated monocytes was assessed with flow cytometry. Data shown are representative of 3 biologically independent experiments. Purity of monocytes was 89.30 ± 1.332% (mean ± SEM). Data shown are representative of 3 biologically independent experiments. (**c**) Decrease of Aβ40 / Aβ42 level in plasma after Aβ40 / Aβ42 removal by immunoprecipitation of Aβ40 / Aβ42 protein with monoclonal antibodies. Data shown are representative of 3 biologically independent experiments. Data are presented as mean values ± SEM with the indicated significance (compared with the HC serum without antibodies application by one-way ANOVA followed by Tukey's post-test). (**d-e**) Aβ40 or Aβ42 was treated to monocytes or monocyte-derived macrophages (20μg/ml, 6h). Migrasome productivity upon Aβ40 (**d**) or Aβ42 (**e**) treatment was evaluated with immunostaining of WGA (red) alone or with TSPAN4 (green). Data are presented as mean values ± SEM with the indicated significance (**d**), and no statistically significant differences were observed upon Aβ42 treatment (**e**) (by two-tailed Student's *t* test). Data shown are representative of 3 biologically independent experiments and migrasomes of 30 cells are calculated. Source data are provided as a Source Data file.

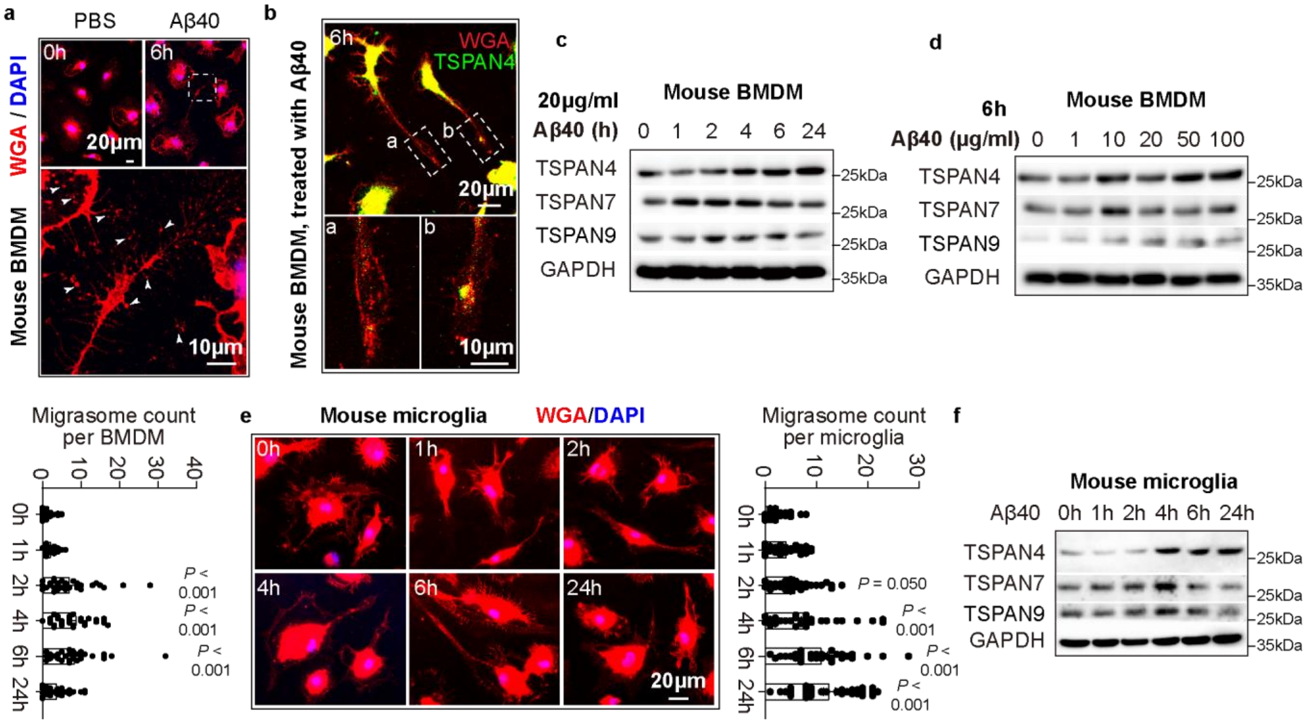

Figure S5. Migrasome productivity of mouse macrophage and microglia upon Aβ40 stimulation. (**a-d**) Mouse bone marrow derived macrophages (BMDM) were cultured and treated with Aβ40. (**a-b**) Migrasome production in BMDM upon Aβ40 stimulation (20μg/ml) was evaluated with immunostaining of WGA (red) alone (**a**) or with TSPAN4 (green) (**b**). Representative confocal images were displayed. Data shown are representative of 3 biologically independent experiments and migrasomes of 30 cells are calculated. Data are presented as mean values ± SEM with the indicated significance (compared with the 0h group by one-way ANOVA followed by Tukey's post-test). (**c-d**) Expression of migrasome markers by BMDM upon Aβ40 treatment for indicated time period (20μg/ml) (**c**) or indicated concentration (6h) (**d**) was assessed with western blot. Data shown are representative of 3 biologically independent experiments. (**e-f**) Mouse microglia were isolated from pups of WT C57/BL6 mice. (**e**) Migrasome production was assessed with immunostaining of WGA upon Aβ40 treatment (20μg/ml) for indicated time points. Data shown are representative of 3 biologically independent experiments and migrasomes of 30 cells are calculated. Data are presented as mean values ± SEM with the indicated significance (compared with the 0h group by one-way ANOVA followed by Tukey's post-test). (**f**) Expression of migrasome markers in mouse microglia upon Aβ40 treatment (20μg/ml) for indicated time points was assessed with western blot. Data shown are representative of 3 biologically independent experiments. Source data are provided as a Source Data file.

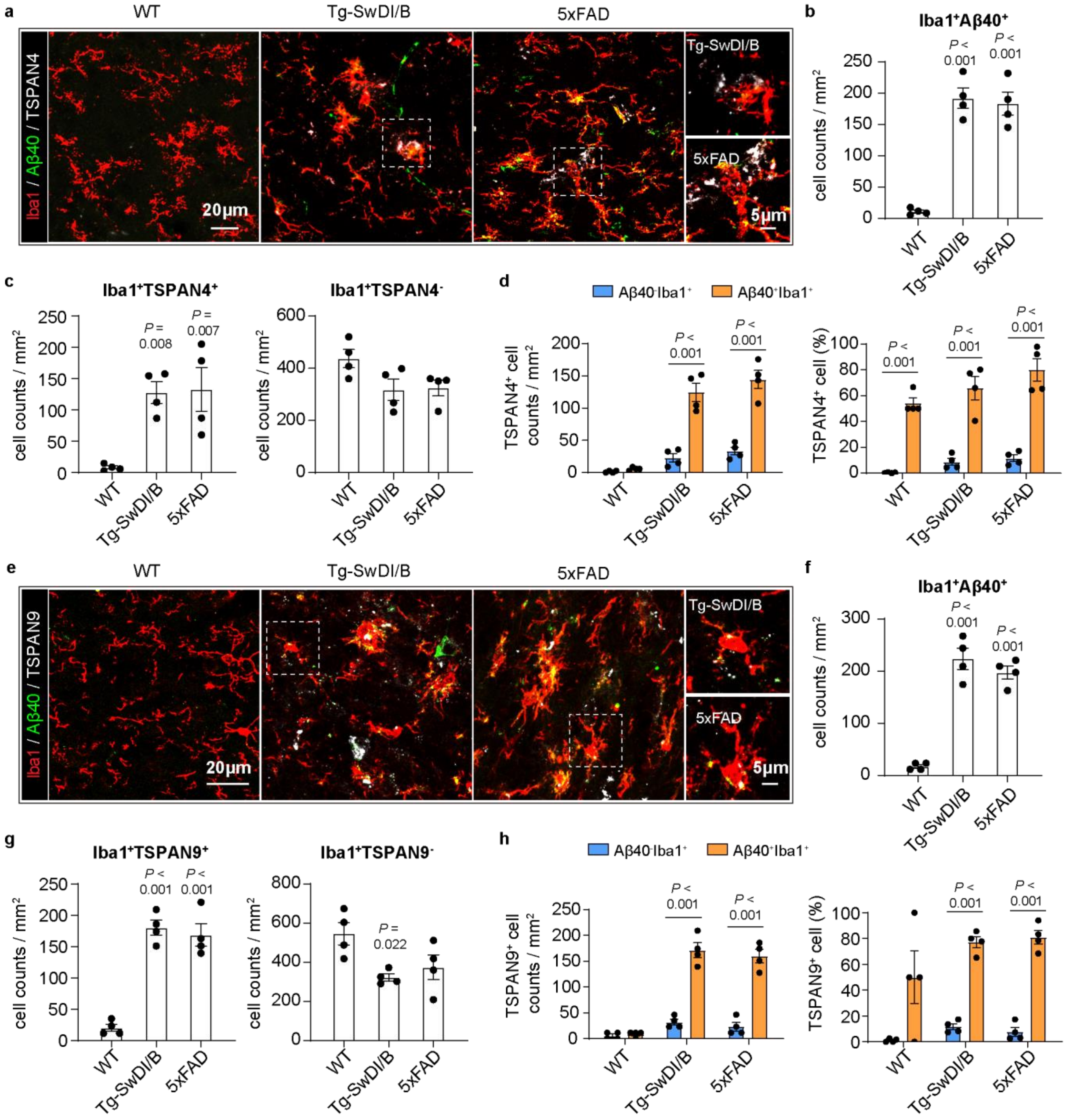

Figure S6. Macrophage lineage cells produce migrasomes when encountering Aβ40 in CAA. Coronal brain sections from Tg-SwDI/B$^{+/+}$ and 5xFAD mice or age- and sex-matched WT mice (24w of age, 2 male and 2 female in each group) were collected and subjected to immunostaining of Iba1/Aβ40/TSPAN4 (**a-d**) or Iba1/Aβ40/TSPAN9 (**e-h**). Aβ40 clearance (Iba1$^+$Aβ40$^+$, **b** and **f**), TSPAN4 (**c**) or TSPAN9 (**g**) expression (Iba1$^+$TSPAN4$^+$ or Iba1$^+$TSPAN9$^+$) and the relationship between Aβ40 engulfment and migrasome production (TSPAN4$^+$Aβ40$^+$Iba1$^+$, **d** or TSPAN9$^+$Aβ40$^+$Iba1$^+$, **h**) in microglia/macrophage were calculated. Data shown are representative of 4 biologically independent experiments. Data are presented as mean values ± SEM with the indicated significance (**b-c, f-g**: compared with WT by one-way ANOVA

followed by Tukey's post-test; **d, h**: by two-tailed Student's *t* test). Source data are provided as a Source Data file.

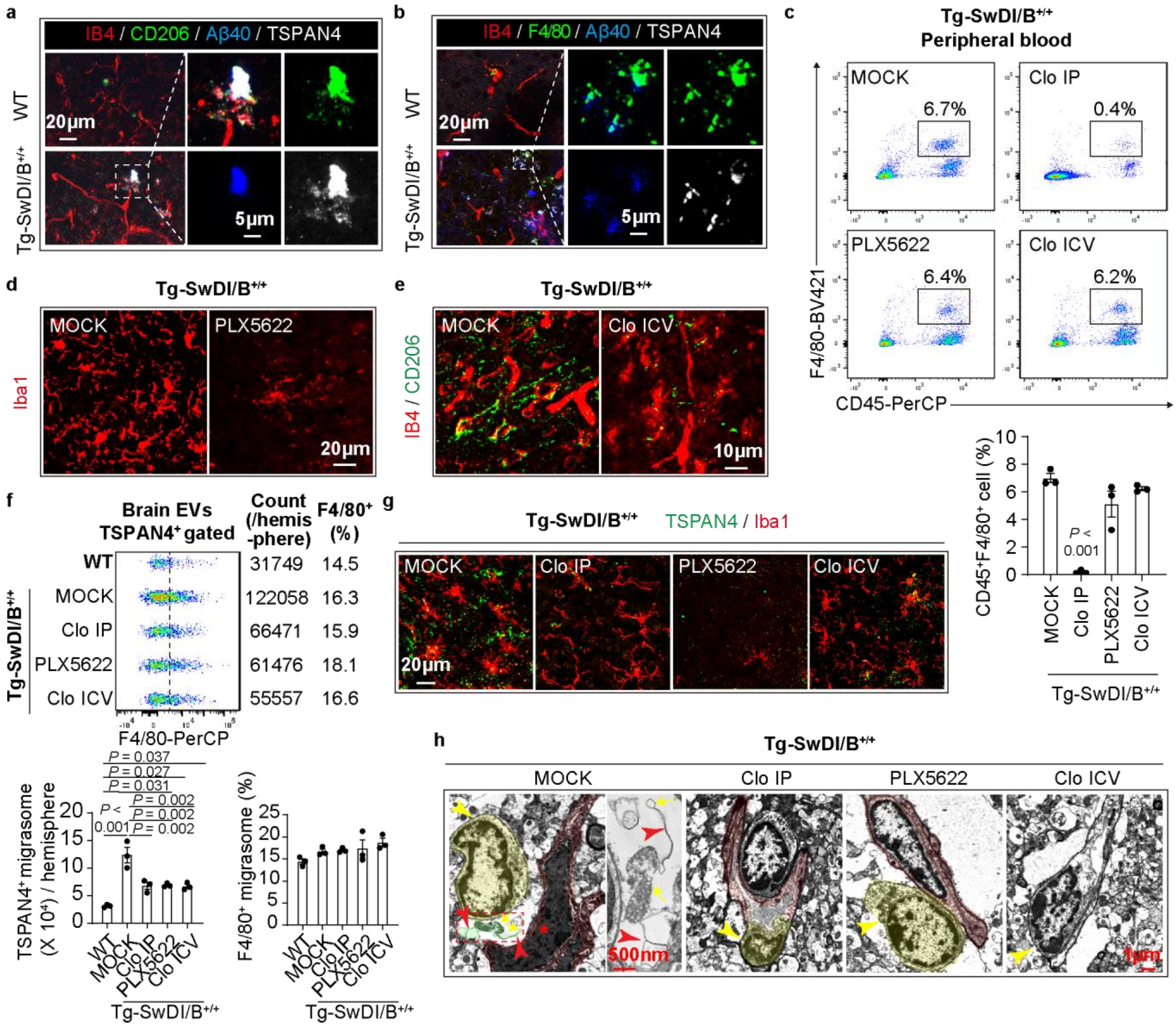

Figure S7. Origin of migrasomes among brain macrophage lineage cells. (**a-b**) Age- and sex-matched WT and Tg-SwDI/B$^{+/+}$ were sacrificed at 24w of age. Coronal brain sections were subjected to immunostaining of lectin (IB4, red), Aβ40 (blue), TSPAN4 (grey) and CD206 (green, PVM marker, **a**) or F4/80 (green, infiltrated macrophages marker, **b**) Representative images showing migrasome produced by PVM or infiltrated macrophages. Data are representative of 3 biologically independent experiments. (**c-h**) Microglia, PVM or circulating monocytes were depleted respectively in Tg-SwDI/B mice (12w) by feed containing the CSF1R inhibitor PLX5622, intra-cerebral ventricle injected clodronate liposomes (Clo ICV) or intraperitoneally injected clodronate liposomes (CLO IP). N = 3 in each group. Depletion efficiency and the impact on other macrophage subsets were assessed with flow cytometry (**c**) or immunostaining (**d-e**). Data are presented as mean values ± SEM with the indicated significance (compared with non-treated mice by one-way ANOVA). Migrasome deposition in brain after depletion of macrophage subsets was analyzed with flow cytometry (**f**), immunostaining (**g**) and TEM (**h**). Data are presented as mean values ± SEM with the indicated significance

(by one-way ANOVA followed by Tukey’s post-test). Data are representative of 3 biologically independent experiments. Source data are provided as a Source Data file.

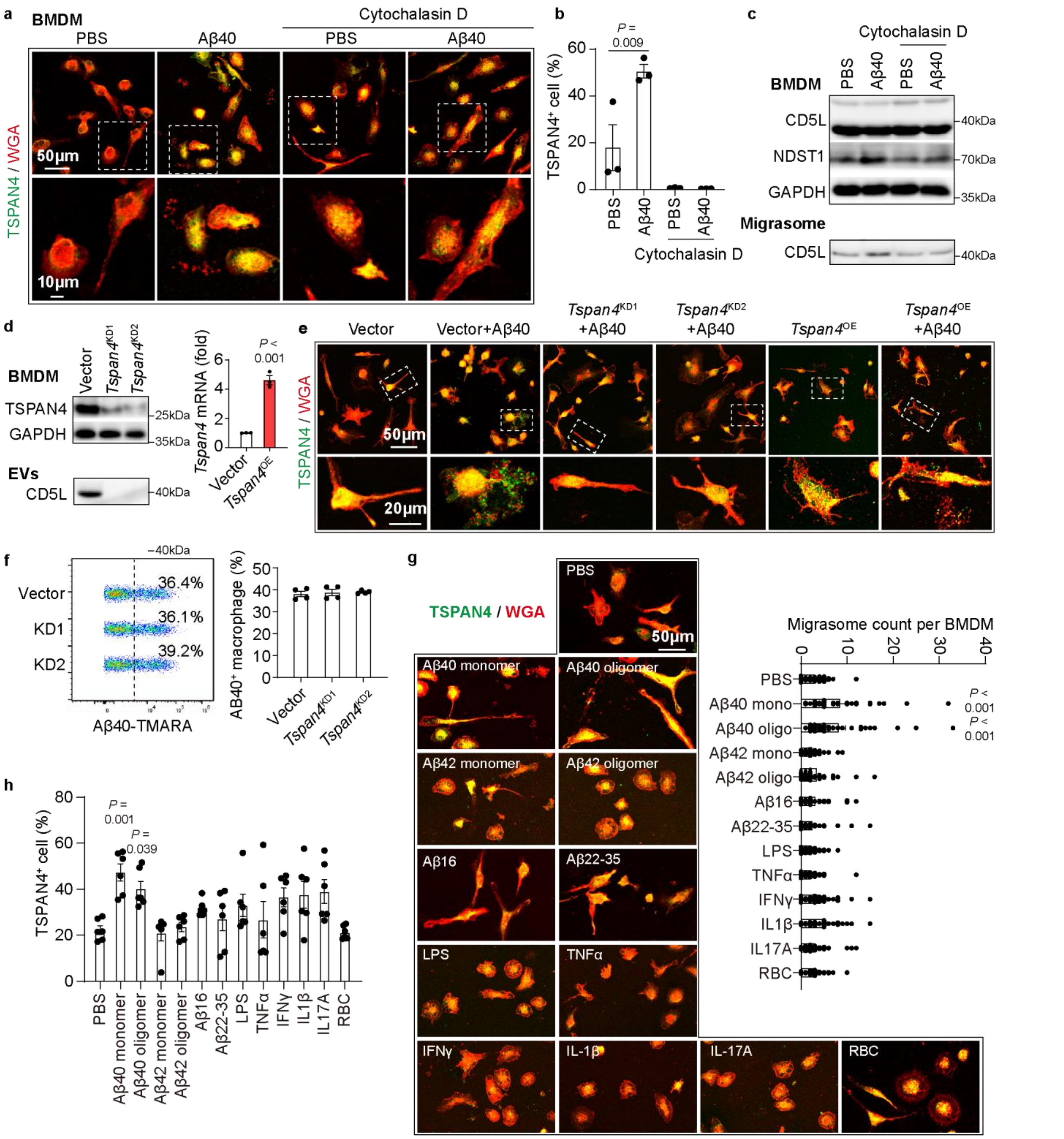

Figure S8. Phagocytosis of Aβ40 specifically elicits migrasome production in macrophages through up-regulating TSPAN4 expression. (**a-c**) Mouse BMDM were pre-treated with Cytochalasin D (CyD, 10μM, overnight) for overnight then stimulated with Aβ40 (20μg/ml, 6h). Data are representative of 3 biologically independent experiments. (**a**) Co-labeling of WGA (red) and TSPAN4 (green). (**b**) TSPAN4 expression in CyD-treated BMDM was assessed with flow cytometry. Data are presented as mean values ± SEM with the indicated significance (by one-way ANOVA followed by Tukey's post-test). (**c**) Expression of CD5L and

NDST1 in the CyD-treated BMDM and CD5L level in the extracellular vesicles (EVs) derived from CyD-treated BMDM as revealed by western blot. (**d-f**) TSPAN4 was over-expressed (OE) or knocked-down (KD) in BMDM. (**d**) **Left**: western blot that displayed the knock-down efficiency of TSPAN4 and CD5L expression in TSPAN4 KD EVs. **Right**: *Tspan4* mRNA level as assessed with QPCR. Data are representative of 3 biologically independent experiments. Data are presented as mean values ± SEM with the indicated significance (by two-tailed Student's *t* test). (**e**) WGA (red) labeling to show the migrasome productivity. Data are representative of 3 biologically independent experiments. (**f**) Phagocytosis of Aβ40 (20μg/ml, 1h) by TSPAN4 KD BMDM as assessed with flow cytometry. Data are representative of 4 biologically independent experiments and presented as mean values ± SEM, and no statistically significant differences were observed (by one-way ANOVA followed by Tukey's post-test). (**g-h**) The following stimulators were treated to BMDM for 6h respectively: Aβ40 monomer (20μg/ml), Aβ40 oligomer (20μg/ml), Aβ42 monomer (20μg/ml), Aβ42 oligomer (20μg/ml), Aβ16 monomer (20μg/ml), Aβ22-35 monomer (20μg/ml), LPS (100ng/ml), TNFα (20ng/ml), IFNγ (20ng/ml), IL-1β (20ng/ml), IL-17A (20ng/ml) and red blood cells (RBC) (BMDM : RBC = 1 : 5). (**g**) Co-labeling of WGA (red) and TSPAN4 (green) of BMDM. (**h**) Expression of TSPAN4 in the stimulated BMDM was assessed with flow cytometry. Data are representative of 6 biologically independent experiments (**h**) and migrasomes of 30 cells are calculated (**g**). Data are presented as mean values ± SEM with the indicated significance (by one-way ANOVA followed by Tukey's post-test). Source data are provided as a Source Data file.

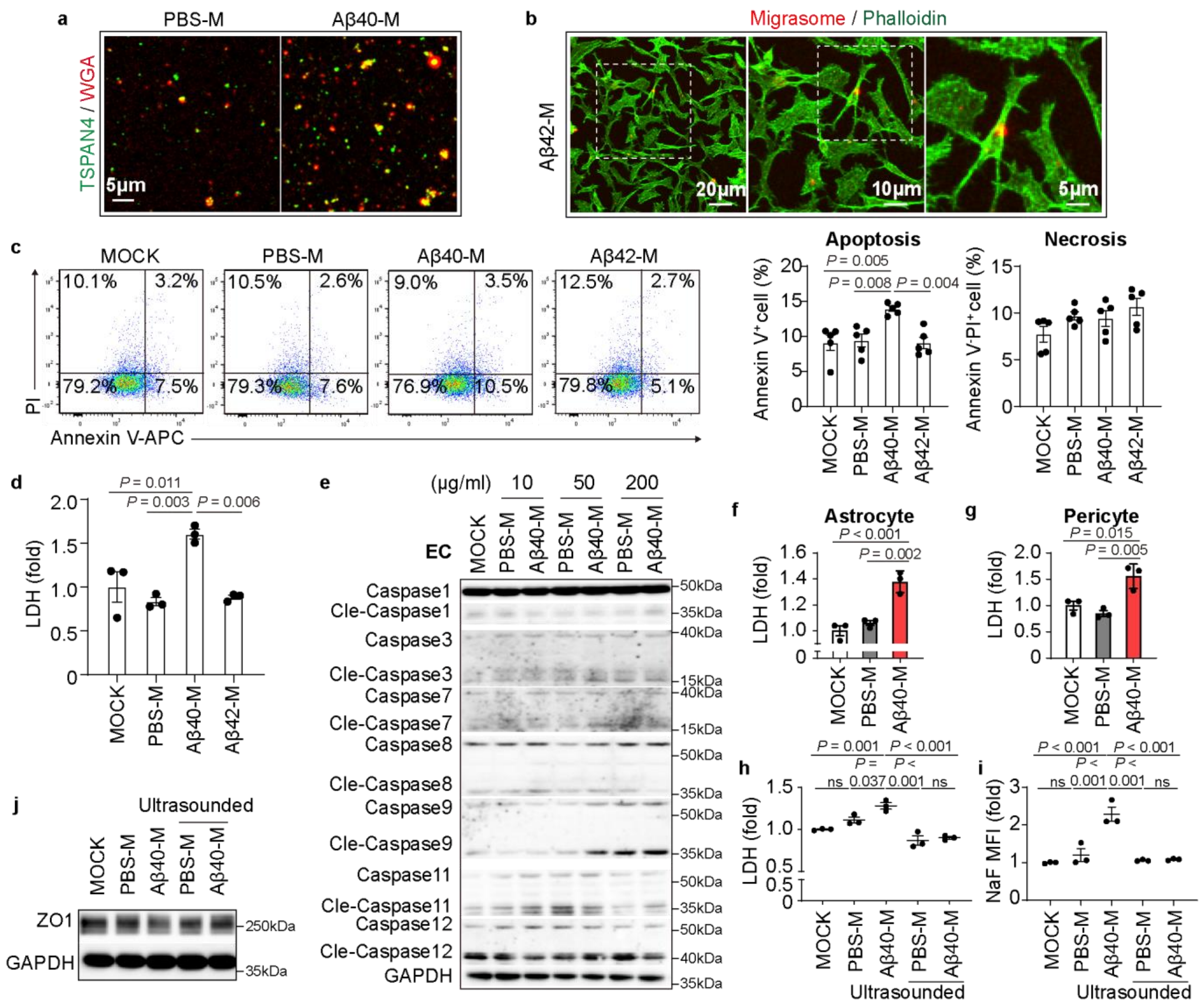

Figure S9. Migrasomes derived from macrophages upon Aβ40 stimulation are injurious to BBB components which is dependent on the migrasome construction. (**a**) Purified PBS-M and Aβ40-M labeled with TSPAN4 (green) and WGA (red). Data are representative of 3 biologically independent experiments. (**b**) Migrasomes derived from Aβ42-treated BMDM (Aβ42-M) were labeled with Dil (red) before treated to endothelial cells (labeled with Phalloidin, green). Adherence of Aβ42-M to endothelial cells was shown with confocal images. Data shown are representative of 3 biologically independent experiments. (**c-d**) PBS-M, Aβ40-M or Aβ42-M were treated to brain blood vessel endothelial cells (50μg/ml, 2h). (**c**) Endothelial injury as assessed with flow cytometric analysis of Annexin V / PI. Data shown are representative of 5 biologically independent experiments. Data are presented as mean values ± SEM with the indicated significance (by one-way ANOVA followed by Tukey's post-test). (**d**) LDH release of the migrasome-treated endothelial cells. Data shown are representative of 3 biologically independent experiments. Data are presented as mean values ± SEM with the indicated significance (by one-way ANOVA followed by Tukey's post-test). (**e**) PBS-M or Aβ40-M were treated to endothelial cells at indicated concentration for 2h. Activation of Caspase signalings

was evaluated with western blot. Data are representative of 3 biologically independent experiments. (**f-g**) PBS-M or Aβ40-M were treated to mouse astrocytes or brain blood vessel pericytes (50μg/ml, 2h). Cell viability of astrocytes (**f**) and pericytes (**g**) was evaluated with LDH release measurement. Data shown are representative of 3 biologically independent experiments. Data are presented as mean values ± SEM with the indicated significance (by one-way ANOVA followed by Tukey's post-test). (**h-j**) Migrasomes with intrinsic structure destroyed by ultrasound (600w, 10min, 3s on/off) were treated to brain vessel endothelial cells (50μg/ml, 2h). LDH release (**h**), permeability to NaF (**i**) and ZO-1 expression (**j**) of the endothelial cells were assessed. Data shown are representative of 3 biologically independent experiments. Data are presented as mean values ± SEM with the indicated significance (by one-way ANOVA followed by Tukey's post-test). Source data are provided as a Source Data file.

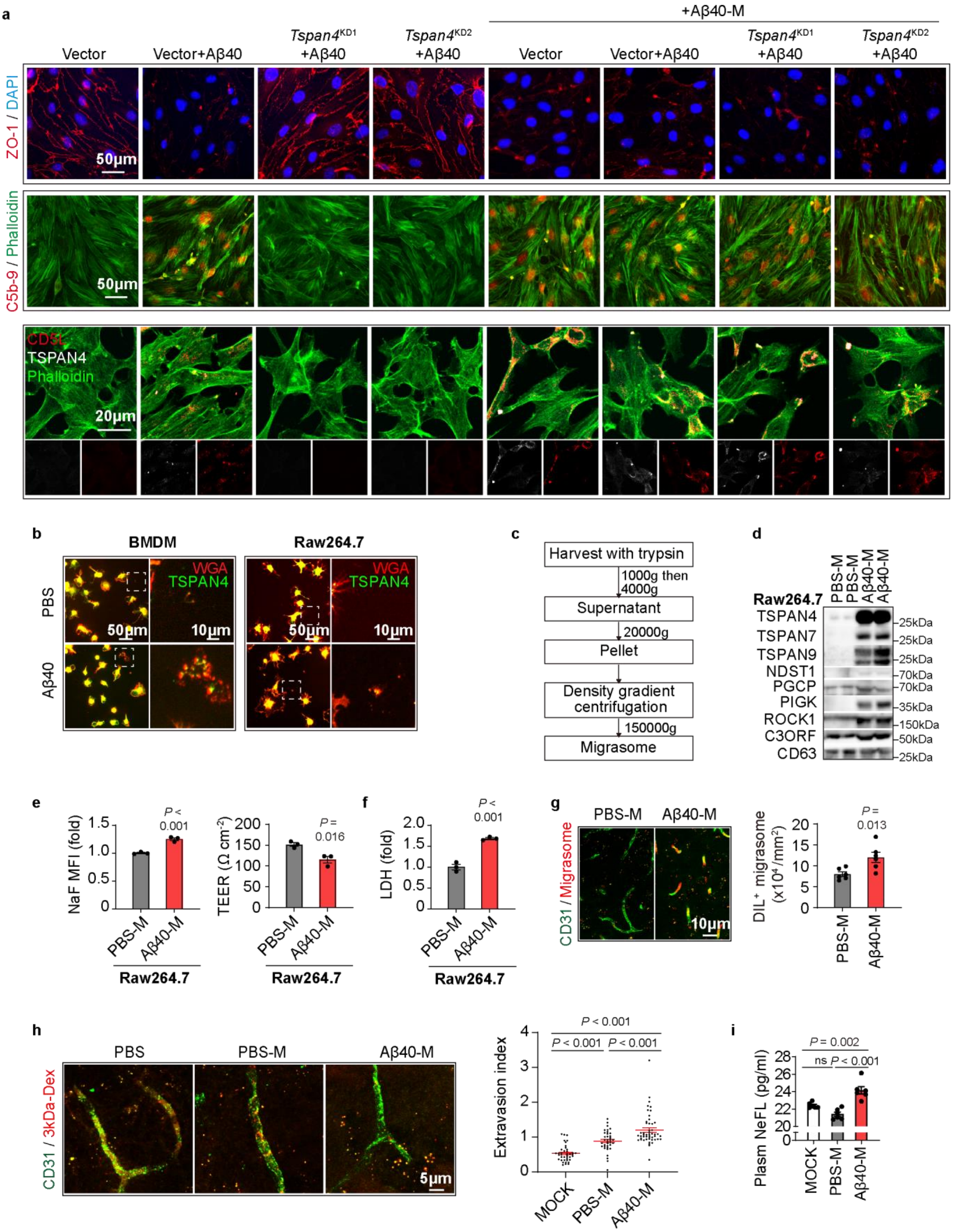

Figure S10. Macrophage-derived migrasomes are adhesive to brain blood vessels and directly damage BBB integrity. (a) ZO-1 expression of endothelial cells, complement activation (C5b-9^{+}) and CD5L deposition were assessed with immunostaining. Data shown are representative of 3 biologically independent

experiments. (**b**) Migrasome production was assessed with WGA and TSPAN4 staining. Data shown are representative of 3 biologically independent experiments. (**c**) Procedure of migrasome collection. (**d**) Expression of migrasome markers in the collected Raw264.7 cell-derived extracellular vesicles was assessed by western blot. Data shown are representative of 3 biologically independent experiments. (**e-f**) Permeability to NaF, the TEER of the barrier, and LDH released by the endothelial barrier were assessed. Data shown are representative of 3 biologically independent experiments and presented as mean values ± SEM with the indicated significance (by two-tailed Student's *t* test). (**g-i**) Healthy C57/BL6 WT mice (age = 8-10w) were treated with 4 serial doses of Raw264.7 cell derived migrasomes (10mg/kg, i.v. injected at day 1, 2, 3, 5). Six days after the first treatment (day 7), recipients were injected with 10mg/kg 3kDa-Dextran at 90min before sacrifice. *N* = 6 in each group. (**g**) Migrasomes were labeled with Dil before injection. Immunolabeling with CD31 (green) of the coronal brain sections of the recipients. Migrasomes attached to the BBB or perfused into the brain parenchyma of recipients had been quantified. Data are presented as mean values ± SEM with the indicated significance (by two-tailed Student's *t* test). (**h**) Extravasation index of 3kDa-Dextran in brain parenchyma of the recipients was calculated as the ratio of extra- to intra-vessel 3kDa-Dextran MFI which was further normalized to that of MOCK group. MFI of 3kDa-Dextran of 40 vessels in MOCK group, 37 vessels in PBS-M transferred group and 49 vessels in Aβ40-M transferred group was calculated. Data are presented as mean values ± SEM with the indicated significance (by one-way ANOVA followed by Tukey's post-test). (**i**) Plasma NeFL level was assessed with ELISA. Data are presented as mean values ± SEM with the indicated significance (by one-way ANOVA followed by Tukey's post-test). Source data are provided as a Source Data file.

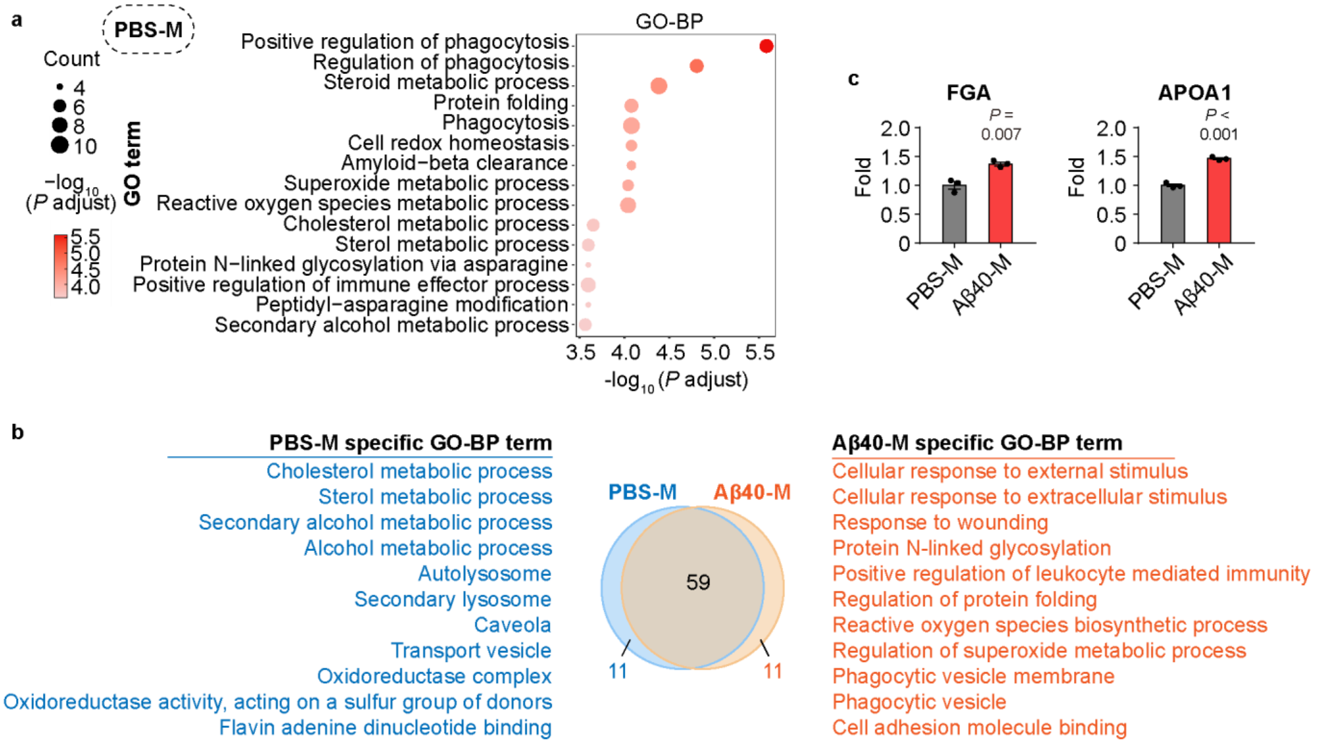

Figure S11. Macrophage-derived migrasomes participate in metabolic regulation in physiological context which is diminished upon Aβ40 stimulation. BMDMs derived migrasomes were collected and subjected to ITRAQ. N = 3 in both groups. (**a**) GO-BP analysis of the identified proteins in PBS-M. (**b**) Proteins identified in PBS-M and Aβ40-M were subjected to Gene Ontology-biological process (GO-BP) projection respectively. Intersection analysis of the enriched GO terms was further performed. (**c**) Expression of cell adhesion molecules FGA and APOA1 as assessed with ITRAQ. Data are presented as mean values ± SEM with the indicated significance (by two-tailed Student's t test). Source data are provided as a Source Data file.

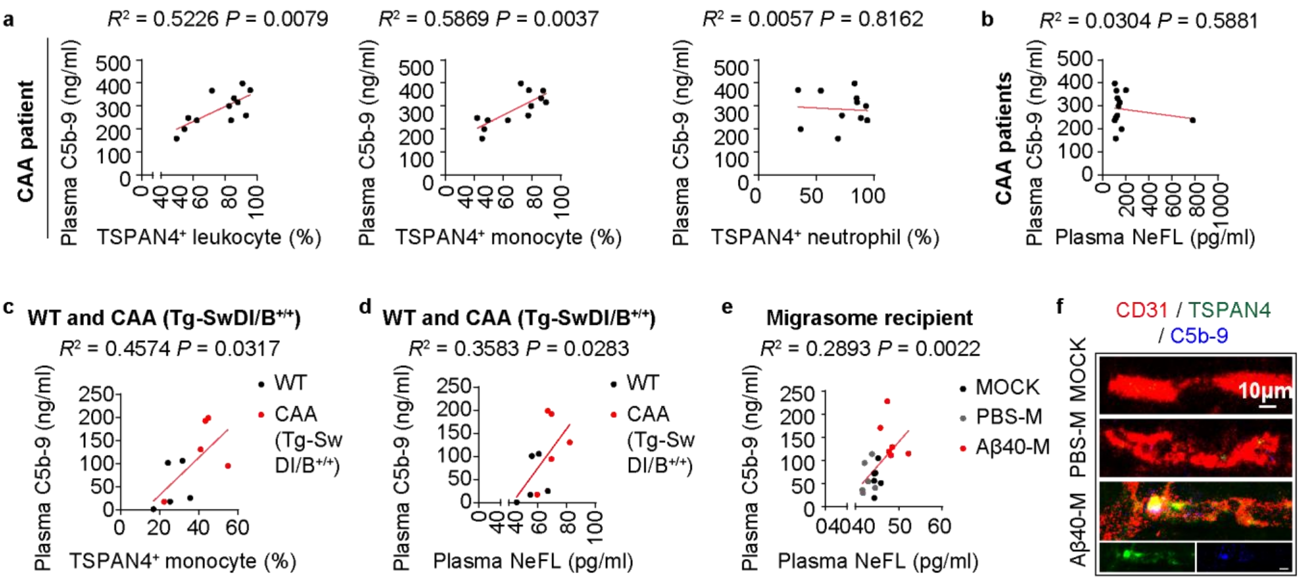

Figure S12. Excessive migrasome productivity of macrophages contributes to complement activation and BBB impairment in CAA. (**a-b**) Peripheral blood of CAA patients (N = 12) and healthy donors (N = 12) were collected. Plasma C5b-9 and NeFL concentration was assessed with ELISA. TSPAN4 expression in total leukocytes, monocytes ($CD14^+$) and neutrophils ($CD66b^+$) was analyzed with flow cytometry. Association between CDC (represented with plasma C5b-9 concentration) with TSPAN4 expression of leukocytes (**a, left**), monocytes (**a, middle**) and neutrophils (**a, right**), or plasma NeFL level (**b**) was estimated with Spearman correlation analysis. (**c-d**) Age- and sex-matched WT and Tg-SwDI/$B^{+/+}$ mice were sacrificed at 24w of age and peripheral blood was collected (N = 5 in each group). Plasma C5b-9 and NeFL concentration was assessed with ELISA. TSPAN4 expression in monocytes ($CD45^+F4/80^+$) was analyzed with flow cytometry. Association between CDC with TSPAN4 expression of monocytes (**c**) or plasma NeFL level (**d**) was estimated with Spearman correlation analysis. (**e-f**) Healthy C57/BL6 WT mice (age = 8-10w) were treated with 4 serial doses of migrasomes (10mg/kg, i.v. injected at day 1, 2, 3, 5). Mice were sacrificed at day 7 and peripheral blood samples were collected. N = 6 in each group. (**e**) Plasma C5b-9 and NeFL concentration was assessed with ELISA. Association between CDC with plasma NeFL level was estimated with Spearman correlation analysis. (**f**) Coronal brain sections of the recipients were collected and subjected to immunostaining of CD31 (red), TSPAN4 (green) and C5b-9 (blue). Scale bar = 10μm. Data shown are representative of 4 biologically independent experiments. Representative images of confocal microscopy were presented. Source data are provided as a Source Data file.

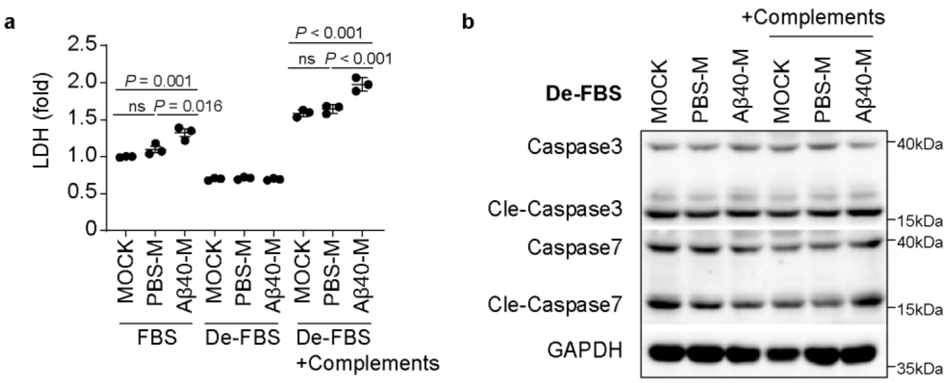

Figure S13. Complement dependent cytotoxicity is indispensable for migrasome mediated blood vessel injury. Brain vessel endothelial cells were treated with 50μg/ml of BMDM-derived migrasomes for 2h with normal FBS, heat-deactivated FBS (65°C, 60min) ± human peripheral blood complement mixture (10% in culture system). LDH release in the culture medium was assessed with ELISA (**a**) and Caspase-3 and -7 activation in the endothelial cells was analyzed with western blot (**b**). Data shown are representative of 3 biologically independent experiments. Data are presented as mean values ± SEM with the indicated significance (by one-way ANOVA followed by Tukey’s post-test). Source data are provided as a Source Data file.

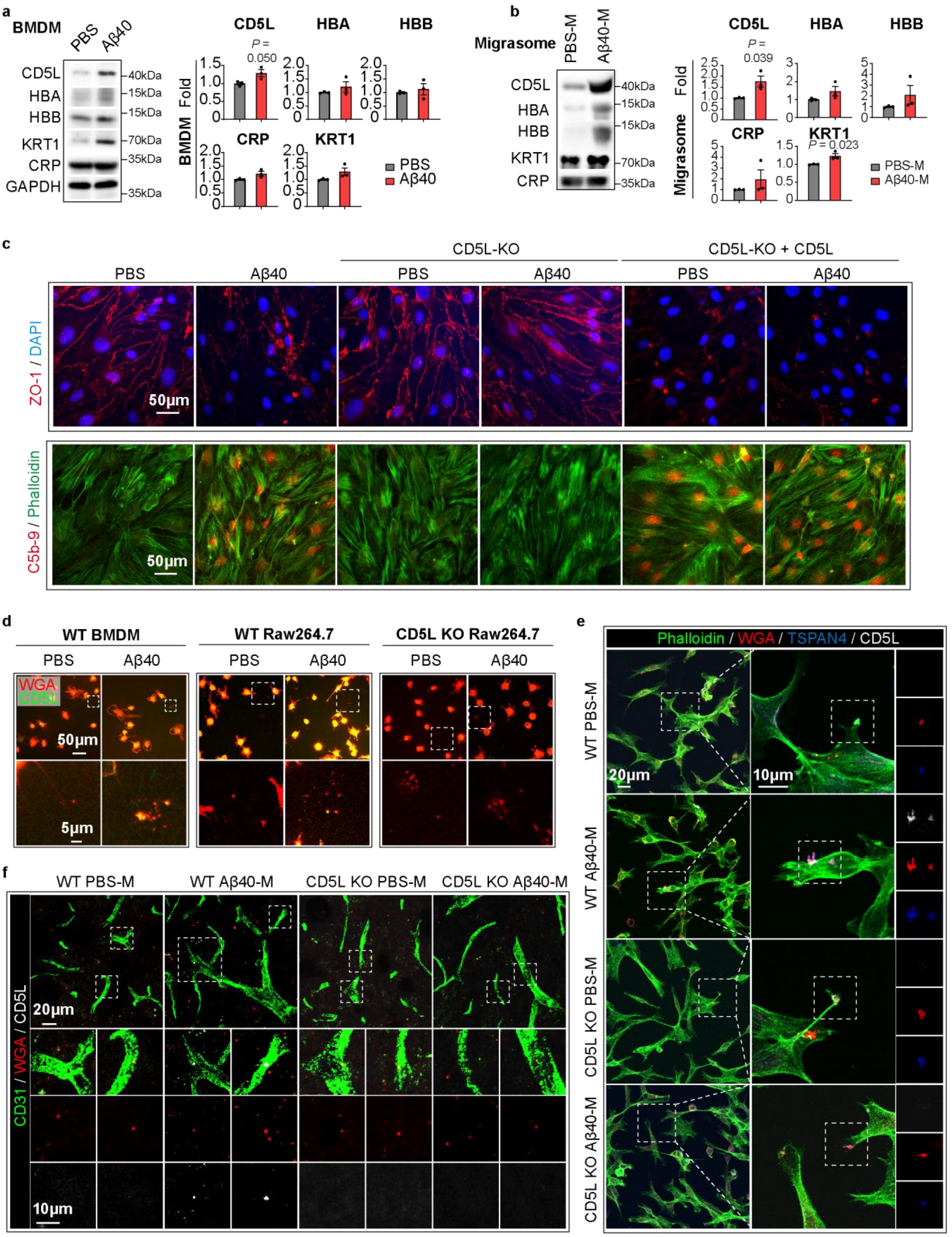

Figure S14. CD5L is enriched in macrophage-derived migrasomes upon Aβ40 stimulation. Mouse BMDM were treated with Aβ40 (50μg/ml, 6h). Expression of the increased complement activation associated proteins in Aβ40-M identified with ITRAQ in BMDM (**a**) and the migrasome product (**b**) was assessed with

western blot. Data shown are representative of 3 biologically independent experiments. Data are presented as mean values ± SEM with the indicated significance (by two-tailed Student's *t* test). (**c**) WT or CD5L KO Raw264.7 cells were stimulated with Aβ40 (20μg/ml, 6h). The Raw264.7 cells (upper chamber) were then coculutred with brain blood vessel endothelial cells (lower chamber) in a transwell based system (2h). ZO-1 expression of endothelial cells and complement activation (C5b-9$^+$) were assessed with immunostaining. Data shown are representative of 3 biologically independent experiments. (**d**) WT BMDM, WT Raw264.7 and or CD5L KO Raw254.7 cells were stimulated with Aβ40 (20μg/ml, 6h). Migrasome production and the presence of CD5L as cargo was assessed with immunostaining. Data shown are representative of 3 biologically independent experiments. (**e**) Migrasomes produced by WT or CD5L KO Raw264.7 cells were collected and stained with WGA. The WGA labeled migrasomes were treated to brain blood vessel endothelial cells (50μg/ml, 2h). Attachment of migrasomes (WGA$^+$TSPAN4$^+$) to endothelial cells (Phalloidin outlined) and release of CD5L were assessed with confocal microscopy. Data shown are representative of 3 biologically independent experiments. (**f**) WT or CD5L KO Raw264.7 cells were stimulated with Aβ40 (20μg/ml, 6h). Migrasomes produced by Raw264.7 cells were collected and stained with WGA. The WGA labeled migrasomes were transferred to healthy C57/BL6 WT mice (age = 8-10w) (10mg/kg, *i.v.* injected at day 1, 2, 3, 5). Six days after the first treatment (day 7), recipients were sacrifice. Presence of the transferred migrasomes (WGA$^+$) in blood vessel (CD31$^+$) and CD5L loaded in the migrasomes were assessed with immunostaining and microscopy. N = 6 in PBS-M, Aβ40-M and CD5L KO Aβ40-M transferred mice and N = 5 in CD5L KO PBS-M treated group. Source data are provided as a Source Data file.

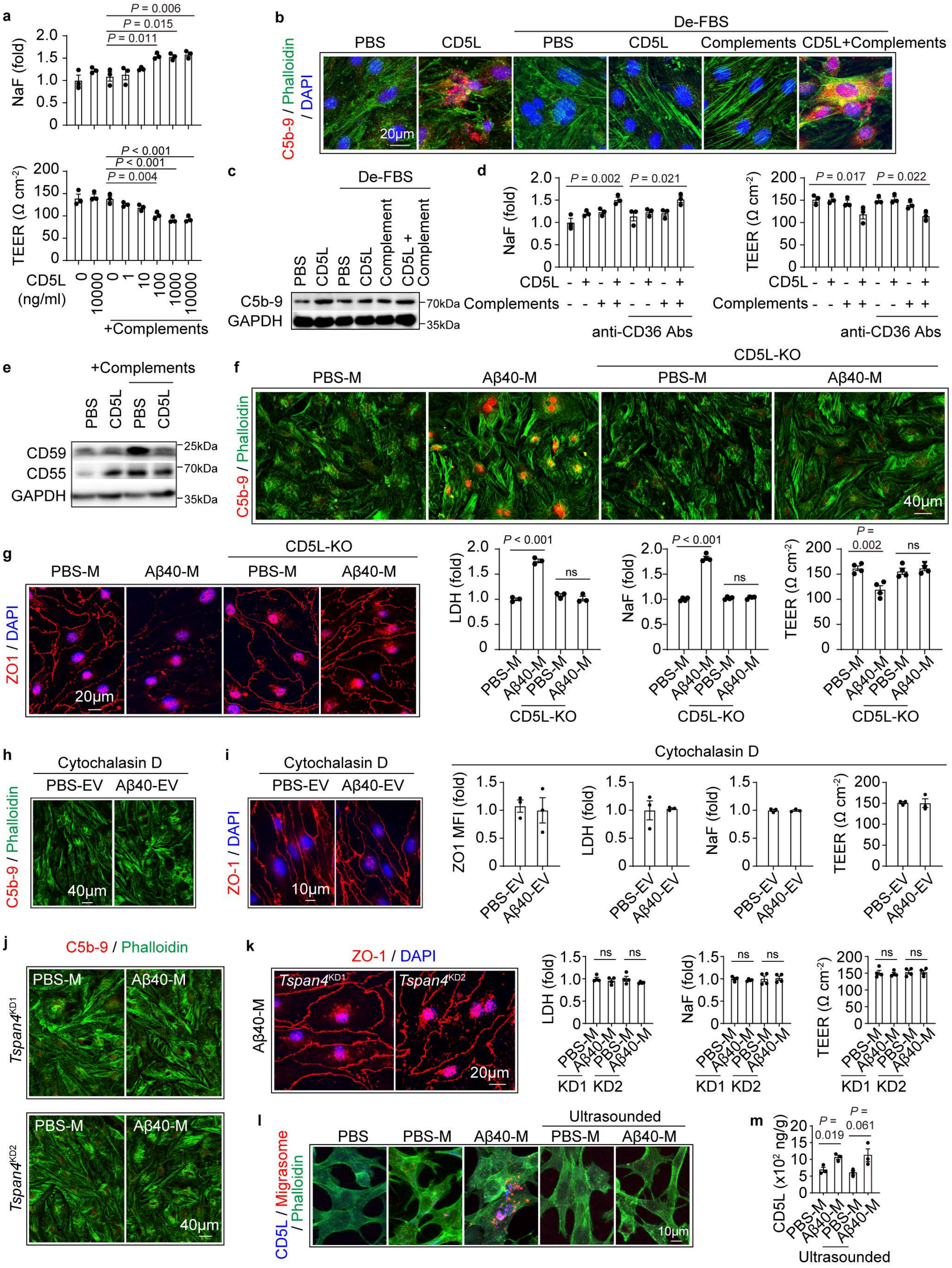

Figure S15. Aβ40-elicited macrophage-derived migrasomes damage BBB through CD5L-associated CDC. (**a-e**) Endothelial cells were cultured with normal or heat-deactivated FBS (65°C, 60min) ± human peripheral blood complement mixture (10%). (**a**) Permeability to NaF and the TEER of the barrier were

assessed at 2h. Data are representative of 3 biologically independent experiments and presented as mean values ± SEM with the indicated significance (by one-way ANOVA followed by Tukey's post-test). (**b-c**) MAC (C5b-9) formation in endothelial cells was assessed with immunostaining (**b**) and western blot (**c**). Data are representative of 3 biologically independent experiments. (**d**) Permeability to NaF and TEER of the barrier were assessed at 2h. Data are representative of 3 biologically independent experiments and presented as mean values ± SEM with the indicated significance (by one-way ANOVA followed by Tukey's post-test). (**e**) Expression of CD59 and CD55 was assessed with western blots. Data are representative of 3 biologically independent experiments. (**f-k**) Aβ40 (20μg/ml) was treated to CD5L KO Raw264.7 macrophages (**f-g**), CyD-treated BMDM (10μM, overnight) (**h-i**) or TSPAN4 KD BMDM (**j-k**) for 6h. MAC (C5b-9) formation in endothelial cells was assessed (**f**, **h**, **j**). Injury of the endothelial barrier was evaluated with the ZO-1 expression, LDH release, permeability to NaF and TEER (**g, i, k**). Data in the LDH release of (**g**), and data in (**i**) are representative of 3 biologically independent experiments. Data in the NaF and TEER experiment of (**g**), and data in (**k**) are representative of 4 biologically independent experiments. Data are presented as mean values ± SEM with the indicated significance (by one-way ANOVA followed by Tukey's post-test (**g** and **k**) and two-tailed Student's *t* test (**i**)). (**l**) Immunostaining of CD5L and phalloidin was performed. Data shown are representative of 3 biologically independent experiments. (**m**) CD5L concentration in Aβ40-M and PBS-M was assessed with ELISA. Data are representative of 3 biologically independent experiments. Data are presented as mean values ± SEM with the indicated significance (by one-way ANOVA followed by Tukey's post-test). Source data are provided as a Source Data file.

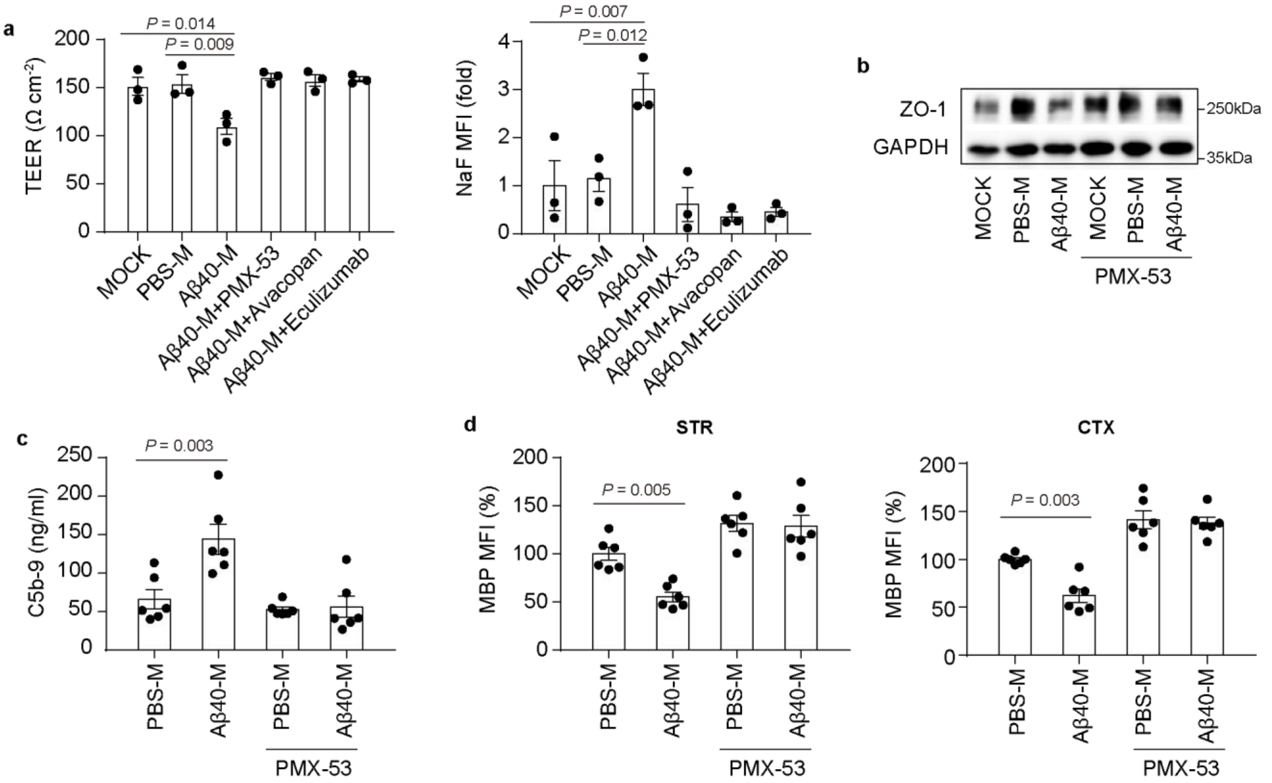

Figure S16. Complement inhibitory treatment reverses the injurious effects of macrophage-derived migrasomes to brain blood vessels. (**a**) Brain vessel endothelial cells were seeded on top of a cell culture insert (pore diameter = 0.4μm) as a simulation of endothelial barrier. Migrasomes (50μg/ml) were treated to the endothelial barrier in addition with PMX-53 (10μg/ml), Avacopan (2μM) or Eculizumab (100μg/ml). Permeability to NaF and TEER of the barrier were assessed at 2h. Data shown are representative of 3 biologically independent experiments. Data are presented as mean values ± SEM with the indicated significance (by one-way ANOVA followed by Tukey's post-test). (**b**) Migrasomes (50μg/ml) were treated to the brain blood vessel endothelial cells in addition with PMX-53 (10μg/ml) for 2h. ZO-1 expression of the endothelial cells was assessed with western blot. Data shown are representative of 3 biologically independent experiments. (**c-d**) Healthy C57/BL6 WT mice (age = 8-10w) were treated with 4 serial doses of Raw264.7 cell derived migrasomes (10mg/kg, i.v. injected at day 1, 2, 3, 5) in addition with PMX-53 treatment (0.25mg per mouse, o.p.). Mice were sacrificed at day 7 after first migrasome injection. (**c**) Plasma C5b-9 level of the recipients was assessed with ELISA. *N* = 6 in each group. Data are presented as mean values ± SEM with the indicated significance (by one-way ANOVA followed by Tukey's post-test). (**d**) Myelin integrity of recipients was assessed with MBP immunostaining. Representative confocal images were displayed in **Figure 7e.** MBP MFI in striatum (STR) and cortex (CTX) was calculated. *N* = 6 in each group. Data are presented as mean values ± SEM with the indicated significance (by one-way ANOVA followed by Tukey's post-test). Source data are provided as a Source Data file.

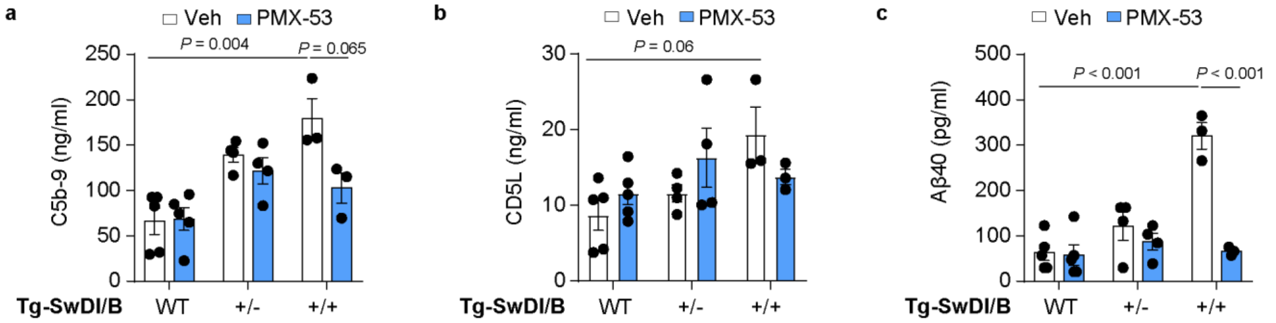

Figure S17. Complement inhibitory therapy protects against CAA without altering plasma Aβ40 and CD5L level. Tg-SwDI/B$^{+/+}$, Tg-SwDI/B$^{+/-}$ and WT mice were treated with PMX-53 (0.25mg per mouse, o.p., for 4 weeks). Mice were sacrificed at 12w of age. Plasma concentration of C5b-9 (**a**), CD5L (**b**) and Aβ40 (**c**) was assessed with ELISA. *N* = 6 in each WT ± PMX-53 groups, *N* = 4 in Tg-SwDI/B$^{+/-}$ ± PMX-53 groups; *N* = 3 in Tg-SwDI/B$^{+/+}$ ± PMX-53 groups. Data are presented as mean values ± SEM with the indicated significance (PMX53 treated Tg-SwDI/B$^{+/+}$ group compared with vehicle treated Tg-SwDI/B$^{+/+}$ group: by two-tailed Student's *t* test; vehicle-treated Tg-SwDI/B$^{+/+}$ group compared with vehicle-treated WT group: by one-way ANOVA followed by Tukey's post-test. Source data are provided as a Source Data file.

Supplementary Tables

Table S1. Clinic characteristics of the individuals recruited in the study.

**Clinical Characteristics**	**Healthy control** ***N*** **=12**	**CAA** ***N*** **=12**	**AIS** ***N*** **=10**	**CADASIL** ***N*** **=10**	**aCSVD** ***N*** **=28**	**AD** ***N*** **=10**
Age, y	70.67±1.13	74.67±2.56	76.30±2.05	46.80±4.49	71.00±1.51	73.10±1.29
Male, *n* (%)	6 (50.00%)	8 (66.67%)	4 (40.00%)	6 (60.00%)	19 (67.86%)	5 (50.00%)
Hypertension, *n* (%)	0	6 (50.00%)	5 (50.00%)	2 (20.00%)	20 (71.43%)	5 (50.00%)
Diabetes mellitus, *n* (%)	0	3 (25.00%)	1 (10.00%)	2 (20.00%)	7 (25.00%)	1 (10.00%)
Symptomatic stroke, *n* (%)	0	7 (58.33%)	10 (100.00%)	4 (40.00%)	7 (25.00%)	1 (10.00%)
**Medication History, n (%)**						
Antithrombotic therapy	0	3 (25.00%)	0	1 (10.00%)	9 (32.14%)	1 (10.00%)
Statins therapy	0	4 (33.33%)	0	1 (10.00%)	10 (35.71%)	2 (20.00%)
Antihypertensive therapy	0	5 (41.67%)	3 (30.00%)	2 (20.00%)	18 (64.29%)	4 (40.00%)
Antidiabetic therapy	0	3 (25.00%)	1 (10.00%)	0	6 (21.43%)	1 (10.00%)
**Laboratory Variables**						
Cholesterol (mmol/L)	5.18±0.29	4.12±0.30	4.85±0.25	4.18±0.31	4.53±0.22	4.81±0.43
Fasting glucose (mmol/L)	4.82±0.25	5.89±0.40	5.91±0.61	5.33±0.42	5.86±0.27	5.23±0.69
Triglyceride (mmol/L)	1.36±0.29	1.26±0.18	1.07±0.11	1.21±0.21	1.50±0.15	108±0.22
HDL-C (mmol/L)	1.95±0.15	1.17±0.10	1.13±0.15	1.19±0.06	1.07±0.07	1.16±0.12
LDL-C (mmol/L)	2.80±0.24	2.38±0.33	3.29±0.22	2.60±0.30	2.74±0.15	3.01±0.40
**Neuropsychological test**						
MoCA	/	10.50 (6.75-18.50)	/	24.50 (19.25-25.00)	21.00 (18.50-23.00)	12.00 (9.50-14.70)
MMSE	/	18.50 (13.75-27.25)	/	28.50 (25.25-29.00)	27.00 (25.00-29.00)	21.50 (19.00-23.00)

Results were presented as mean ± SEM for normally distributed continuous variables and median (quartiles) for skewedly distributed continuous variables. HDL-C, high density lipoprotein cholesterol; LDL-C, low density lipoprotein cholesterol.

Table S2. Imaging characteristics of CSVD patient.

Imaging Characteristics	**CAA *N* =12**	**CADASIL *N* =10**	**aCSVD *N* =28**
Presence of cSS, n (%)	9 (75.00%)	0	0
CMB Counts, medium (quartiles)	2.50 (1.00-8.25)	12.00 (0.50-21.75)	1.00 (0.00-3.25)
d-WHN Fazekas, medium (quartiles)	2.00 (1.00-3.00)	2.00 (2.00-2.00)	1.50 (1.00-2.00)
p-WHN Fazekas, medium (quartiles)	2.00 (1.00-3.00)	2.00 (2.00-3.00)	2.00 (1.00-2.00)
PVS Scores, medium (quartiles)	2.00 (1.00-2.00)	2.00 (2.00-2.00)	2.00 (0.00-3.00)
Lacunar Counts, medium (quartiles)	4.00 (2.75-6.00)	0.00 (0.00-0.75)	1.00 (0.00-2.00)
CAA Burden, medium (quartiles)	3.50 (2.00-5.00)	2.00 (2.00-2.00)	1.00 (0.00-2.00)
CSVD Burden, medium (quartiles)	4.00 (3.00-4.00)	3.00 (2.00-3.75)	2.00 (1.00-3.00)
